# Clinical exome sequencing for fetuses with ultrasound abnormalities and a suspected Mendelian disorder

**DOI:** 10.1186/s13073-018-0582-x

**Published:** 2018-09-28

**Authors:** Elizabeth A. Normand, Alicia Braxton, Salma Nassef, Patricia A. Ward, Francesco Vetrini, Weimin He, Vipulkumar Patel, Chunjing Qu, Lauren E. Westerfield, Samantha Stover, Avinash V. Dharmadhikari, Donna M. Muzny, Richard A. Gibbs, Hongzheng Dai, Linyan Meng, Xia Wang, Rui Xiao, Pengfei Liu, Weimin Bi, Fan Xia, Magdalena Walkiewicz, Ignatia B. Van den Veyver, Christine M. Eng, Yaping Yang

**Affiliations:** 1 Department of Molecular and Human Genetics, Baylor College of Medicine, Houston, TX USA; 2 Baylor Genetics, Houston, TX USA; 3 Human Genome Sequencing Center, Baylor College of Medicine, Houston, TX USA; 4 Present address: The National Institute of Allergy and Infectious Disease, NIH, Bethesda, MD USA; 5 Department of Obstetrics and Gynecology, Baylor College of Medicine, Houston, TX USA

**Keywords:** Fetal structural abnormalities, Exome sequencing, Prenatal, Single-gene disorder, Mendelian disease

## Abstract

**Background:**

Exome sequencing is now being incorporated into clinical care for pediatric and adult populations, but its integration into prenatal diagnosis has been more limited. One reason for this is the paucity of information about the clinical utility of exome sequencing in the prenatal setting.

**Methods:**

We retrospectively reviewed indications, results, time to results (turnaround time, TAT), and impact of exome results for 146 consecutive “fetal exomes” performed in a clinical diagnostic laboratory between March 2012 and November 2017. We define a fetal exome as one performed on a sample obtained from a fetus or a product of conception with at least one structural anomaly detected by prenatal imaging or autopsy. Statistical comparisons were performed using Fisher’s exact test.

**Results:**

Prenatal exome yielded an overall molecular diagnostic rate of 32% (*n* = 46/146). Of the 46 molecular diagnoses, 50% were autosomal dominant disorders (*n* = 23/46), 41% were autosomal recessive disorders (*n* = 19/46), and 9% were X-linked disorders (*n* = 4/46). The molecular diagnostic rate was highest for fetuses with anomalies affecting multiple organ systems and for fetuses with craniofacial anomalies. Out of 146 cases, a prenatal trio exome option designed for ongoing pregnancies was performed on 62 fetal specimens, resulting in a diagnostic yield of 35% with an average TAT of 14 days for initial reporting (excluding tissue culture time). The molecular diagnoses led to refined recurrence risk estimates, altered medical management, and informed reproductive planning for families.

**Conclusion:**

Exome sequencing is a useful diagnostic tool when fetal structural anomalies suggest a genetic etiology, but other standard prenatal genetic tests did not provide a diagnosis.

**Electronic supplementary material:**

The online version of this article (10.1186/s13073-018-0582-x) contains supplementary material, which is available to authorized users.

## Background

Congenital fetal anomalies occur in approximately 3% of pregnancies and are responsible for 20% of infant mortality in the USA [1, 2]. Many of these are thought to have an underlying genetic etiology. Current practice guidelines recommend karyotype and chromosomal microarray analysis (CMA) as first-tier tests when a fetal anomaly has been detected by ultrasound or other fetal imaging [3, 4]. These tests are able to detect aneuploidy, chromosomal rearrangements, or copy number variants (CNVs) in a combined 30–40% of pregnancies studied [5–8].

While these genetic testing approaches are invaluable for prenatal genetic diagnosis, the potential etiology for fetal anomalies remains unsolved in approximately 60% of cases. A proportion of these unsolved cases may be the result of Mendelian disease due to single-gene defects. Historically, clinicians relied on serial sequencing of single genes or gene panels to explore a potential molecular diagnosis for a Mendelian disease trait. However, such approaches usually require a fairly narrow differential diagnosis and are time consuming. This poses a clinical conundrum in prenatal medicine, where the ability to narrow the differential diagnosis may be limited by incomplete phenotypic information due to the inherent limitations of in utero imaging or gestational age. Even when the clinical phenotype manifested during pregnancy is highly specific, targeted gene tests may yield negative results if the disorder is caused by a variant in a disease gene that is not included in the chosen panel.

One solution to this diagnostic challenge is exome sequencing (ES), which has been shown to provide a valuable diagnostic option in postnatal genetic evaluation because it is not disease- or gene-specific and does not require prior knowledge regarding the potential causative gene(s) for an observed phenotype [9]. Exome sequencing has therefore started to be incorporated into clinical care for pediatric and adult populations. While there have been multiple publications showing the diagnostic and clinical utility of ES in the postnatal setting [3, 4, 10–19], integration of ES into prenatal diagnosis has been more limited. One reason for this is the paucity of information about the clinical utility of ES in the prenatal setting [20, 21]. Here we present a retrospective analysis of the outcomes of prenatal ES that was performed in a diagnostic laboratory as part of the clinical management of pregnancies, including continuing pregnancies, complicated by fetal structural anomalies.

## Methods

### Sample inclusion criteria

We performed a retrospective review of the indications, exome results, and clinical impact of molecular diagnoses for all fetal samples that were referred to the Baylor Genetics clinical diagnostic laboratory by a physician for exome testing. We defined the following inclusion criteria: (1) the fetal sample was obtained through an invasive diagnostic procedure (including amniocentesis, chorionic villus sampling, and cordocentesis) or product of conception (POC), (2) the fetus had at least one structural anomaly detected by fetal imaging or autopsy, (3) ES was performed at Baylor Genetics, and (4) a final report was issued between March 2012 and November 2017. The Baylor Genetics clinical diagnostic laboratory is accredited by the College of American Pathologists (CAP) and certified by the US Department of Health and Human Services Clinical Laboratory Improvement Amendments (CLIA). De-identified reporting of demographic and molecular data from this laboratory was approved by the Institutional Review Board at Baylor College of Medicine.

### Consent procedures and testing protocols

All exome tests involving a fetal sample required informed consent from parents, relevant patient clinical data, and prior approval by a laboratory genetic counselor that ES was an appropriate testing option. Fetal exomes were processed under one of three testing protocols: proband exome (turnaround time (TAT): 12 weeks, available since 2011), trio exome (TAT: 8 weeks, available since 2014), or prenatal trio exome (TAT: 2–3 weeks excluding tissue culture time, available since 2015). The prenatal trio exome test was intended specifically for ongoing pregnancies and was therefore designed with specialized consenting and reporting procedures. The prenatal trio exome consent form did not include options to report reproductive carrier status [22] or variants in medically actionable genes [23, 24] for the fetus. These options were available for the parents at the time of exome sequencing and for the proband after birth upon request.

Selection of the appropriate prenatal exome test was affected by the availability of the method and parental samples at the time of testing and the degree of urgency of the case. For proband ES, next-generation sequencing (NGS) was only performed on the fetal sample and those sequencing results were interpreted in the context of the clinical indications (Additional file 1: Figure S1). Sanger sequencing of clinically relevant variants was then performed on the fetal and available parental samples before final variant interpretation and reporting. The standard and prenatal trio exome tests required that both parental samples accompany the fetal sample. For these tests, the fetal and parental samples underwent ES simultaneously and results were interpreted in unison, taking into account de novo variants in the fetal DNA sample, inheritance, and allelic configuration of each variant, as well as the clinical indications (Additional file 1: Figure S1).

### Laboratory exome analysis, variant interpretation and result reporting

Fetal DNA was extracted from chorionic villus samples (CVS), amniotic fluid, tissue samples, or fetal blood. For samples requiring cell culturing before DNA extraction, the TAT was calculated from the date of DNA extraction. DNA was extracted from peripheral blood or saliva samples from both biological parents. Previously extracted DNA from any of these sources was also accepted**.** All fetal samples were tested for maternal cell contamination by comparison of maternal and fetal DNA using AmpFlSTR® Identifiler®, which simultaneously amplifies 15 short tandem repeat sites and a gender-determining marker on sex chromosomes. All exome samples were also concurrently tested by an Illumina HumanOmni1-Quad or HumanExome-12 v1 single nucleotide polymorphism (SNP) array for quality control of the exome data and to detect large CNVs, absence of heterozygosity (AOH), and uniparental disomy.

Exome sequencing was performed on DNA samples as previously described [10, 11]. The following metrics were achieved for all samples: mean depth of coverage was ~ 150×, and ~ 98% of target bases (exons and ± 20 intronic nucleotides flanking the exon-intron boundaries of all nuclear genes) were interrogated at > 20× read depth (Additional file 1: Table S1). Variants were assessed for pathogenicity based on the adapted American College of Medical Genetics and Genomics (ACMG) guidelines [25] by a team of American Board of Medical Genetics and Genomics-certified molecular lab directors and medical directors as previously described [10, 26].

On the fetal report, pathogenic and likely pathogenic variants that may be causative of or related to the prenatal indications were included; variants of unknown significance (VUS) were occasionally included when there was a strong indication for reporting (e.g., the VUS was compound heterozygous with a pathogenic variant). Using similar inclusion criteria, variants likely to cause significant, childhood-onset disorders not related to the prenatal indications were also included on the fetal report; reporting of such incidental findings was done on a case-by-case basis based on a consensus decision between the laboratory and the ordering physician.

A fetal exome sample was classified as molecularly diagnosed if the aforementioned variant(s) were detected in a disease gene that was consistent with the clinical phenotype of the fetus and the expected disease inheritance pattern. For biallelic variants in presumed autosomal recessive disorders, the phase of the variants was assessed by parental studies (by exome in the case of trio exomes, or by Sanger sequencing in the case of proband exomes). The variants were considered to constitute a molecular diagnosis only if they were determined to be in *trans*. In some cases, a pathogenic or likely pathogenic variant in *trans* with a VUS was considered to constitute a molecular diagnosis depending on the phenotypic specificity and overlap with the fetal findings. For dominant disorders, only de novo variants or those that were inherited from a mosaic or affected parent were considered to contribute to a molecular diagnosis. Rarely, it was possible for a VUS or a partial phenotype overlap to contribute to a molecular diagnosis upon consultation with the ordering physician. Clinical impacts and postnatal outcome data were collected for samples that were referred for genetic testing by a local clinical institution.

Initial fetal reports could be issued without Sanger sequencing confirmation if the NGS variant call(s) on the report were of high confidence (coverage ≥ 20×, minor allele fraction ≥ 30%, and Phred score of variant calling ≥ 30).

Fetal phenotype information was converted into top-branch Human Phenotype Ontology (HPO) categories using Phenomizer [27, 28]. Statistical comparisons were performed using the two-tailed Fisher’s test. The Bonferroni correction was applied for multiple comparisons.

## Results

### Sample characteristics

One hundred and forty-six prenatal samples fulfilling the designated clinical inclusion criteria were received for fetal exome testing and had a final report issued. The majority of fetal samples were received as extracted DNA (*n* = 43) or amniotic fluid (*n* = 35 cultured, *n* = 32 direct, Additional file 1: Figure S2). The remaining sample types included POC (*n* = 17 direct, *n* = 7 cultured), cord blood (*n* = 5), and CVS (*n* = 4 direct, *n* = 3 cultured, Additional file 1: Figure S2). To our knowledge, a CMA and/or karyotype was performed prior to exome analysis for 132 of 146 families, but was non-diagnostic for an etiological molecular diagnosis. In two cases, sex chromosome abnormalities were detected (47,XXY and 47,XYY), but because the chromosomal findings did not explain the fetal phenotype, prenatal exome testing was initiated. One sample was referred for exome testing although there was a previously identified CNV of uncertain clinical significance that could potentially explain the fetal anomalies. In this case, ES did not detect additional pathogenic variants, so it was not considered to have a molecular diagnosis in our current analysis. CMA and/or karyotype results for the proband fetus were not available for the remaining 14/146 families. For 6 of these families, a previous similarly affected fetus had non-diagnostic CMA and/or karyotype results, but such analysis of the proband fetus was either in progress, not performed, or results were not provided to our laboratory at the time of testing.

Cases were referred from Genetics (*n* = 67), Maternal and Fetal Medicine (*n* = 45), Obstetrics (*n* = 26), Pediatrics (*n* = 3), Pediatric Neurology (*n* = 2), and Pathology (*n* = 3) departments (Additional file 1: Figure S3). The majority of samples (*n* = 123) were referred from an academic institution, while 23 were from a private institution (Additional file 1: Figure S3).

In addition to the cohort of 146 samples with completed prenatal exome testing, exome testing was not completed for 13 samples (Additional file 1: Table S2). In 6 cases, testing was canceled at the request of the referring institution. In 7 cases, we were unable to issue a final report due to insufficient samples.

### Reported variants, molecular diagnostic rate and TAT

Of 146 total cases, 46 received a molecular diagnosis from exome sequencing, an overall diagnostic rate of 32% (Table 1). Fifty-nine contributing variants, including 8 frameshift, 11 stopgain, 7 splice site, 2 in-frame insertions/deletions, and 31 nonsynonymous changes, were reported in these 46 cases (Table 2). Both parental samples were available for testing in 142 of the 146 cases. Fetal samples in this cohort underwent exome testing by one of three available testing options as described in the “Methods” section: prenatal trio (*n* = 62), standard trio (*n* = 33), or proband exome (*n* = 51). A molecular diagnosis was reported for 35% of prenatal trio exomes (*n* = 22/62), 21% of standard trio exomes (*n* = 7/33), and 33% of proband exomes (*n* = 17/51; Table 1). There was no statistically significant difference in the diagnostic rates of the three groups (prenatal trio versus standard trio, *p* = 0. 370; proband versus all trios, *p* = 0.860). The mean TAT from DNA extraction to initial result reporting was 2.0 weeks (range 1.0–5.4 weeks) for prenatal trio exome, 6.2 weeks (range 1.9–11.1 weeks) for standard trio exome, and 12.6 weeks (range 2.6–20.2 weeks) for proband exome (Table 1). Time required for culturing was excluded from these TAT calculations because an ES test order was received concurrently with the sample for only 22% of samples (*n* = 33/146) for which culturing was required (Cohorts 1a and 1b, Additional file 1: Figure S2). The remaining samples either did not require any culturing (58%, *n* = 84/146) or the ES test was ordered sometime after sample receipt, culture initiation, and/or DNA extraction was complete (20%, *n* = 29/146, Additional file 1: Figure S2).

**Table 1:** Molecular diagnostic rate and turnaround time by test type

Exome type	No. of cases	No. of molecular diagnoses	Diagnostic rate	Mean TAT (range, weeks)
Prenatal trio	62	22	35%	2.0 (1.0–5.4)
Standard trio	33	7	21%	6.2 (1.9–11.1)
Proband	51	17	33%	12.6 (2.6–20.2)
Total	146	46	32%	

The overall molecular diagnostic rate, considering all exome test types, is 32% (*n* = 46/146). The molecular diagnostic rates of each test type (prenatal trio, 35%; standard trio, 21%; proband, 33%) are not significantly different (*p* > 0.05, Fisher’s exact test). The mean turnaround time (TAT) for each test type is indicated and the range is indicated in parentheses

**Table 2:** Fetal molecular diagnoses

Case ID	Gene	Variants [RefSeq ID]	Inheritance/zygosity	Clinical impact	Pre-test recurrence risk	Post-test recurrence risk	Disease association(s) [MIM #]
30-P	*ACTA1*	c.116G>A (p.R39H) [NM_001100]	AD/de novo het	NR	NR	RES	Nemaline myopathy 3 [MIM: 161800]; Myopathy, congenital, with fiber-type disproportion 1 [MIM: 255310]
24-P	*ADGRG6*	c.2677C>T (p.R893X) [NM_020455]	AR/homozygous	Reproductive planning	NR	25%	Lethal congenital contracture syndrome 9 [MIM: 616503]
8-P	*ALG12*	c.437G>A (p.R146Q) c.930_931delAC (p.R311fs) [NM_024105]	AR/compound het	Reproductive planning	NR	25%	Congenital disorder of glycosylation type 1G [MIM: 607143]
111-T	*AR*	c.1814A>G (p.D605G) [NM_000044]	XL/hemizygous (maternally inherited)	Reproductive planning Recurrence risk	Unknown	50% (males)	Complete androgen insensitivity syndrome [MIM: 300068]
43-PRE	*C5orf42*	c.3667C>T (p.Q1223X) c.1372-2A>G [NM_023073*]*	AR/compound het	NR	NR	25%	Orofaciodigital syndrome 6 [MIM: 277170]; Joubert syndrome 17 [MIM: 614615]
87-PRE	*CHRNG*	c.136C>T (p.R46X) c.459dupA (p.V154fs) [NM_005199]	AR/compound het	NR	NR	25%	Multiple pterygium syndrome, lethal type [MIM: 253290]; Multiple pterygium syndrome, Escobar variant [MIM: 265000]
80-T	*COL11A1*	c.2739_2747del (p.P914_G916del) [NM_001854]	AD/de novo het	NR	NR	RES	Stickler syndrome 2 [MIM: 604841]; Marshall syndrome [MIM: 154780]; Fibrochondrogenesis 1 [MIM: 228520]
17-P	*COL1A1*	c.2110G>A (p.G704S) [NM_000088]	AD/de novo het	NR	NR	RES	Osteogenesis imperfecta (OI) types 1–4 [MIM: 166200, 166210, 259420, 166220]; Caffey disease [MIM: 114000]; Ehlers-Danlos syndrome 1 and 7a [MIM: 130000, 130060]
49-T	*COL1A1*	c.2533G>A (p.G845R) [NM_000088]	AD/de novo het	Recurrence risk	Up to 25%	RES	Osteogenesis imperfecta (OI) types 1–4 [MIM: 166200, 166210, 259420, 166220]; Caffey disease [MIM: 114000]; Ehlers-Danlos syndrome 1 and 7a [MIM: 130000, 130060]
90-PRE	*COL1A1*	c.2164G>A (p.G722S) [NM_000088]	AD/de novo het	Medical management Reproductive planning Recurrence risk	Up to 25%	RES	Osteogenesis imperfecta (OI) types 1–4 [MIM: 166200, 166210, 259420, 166220]; Caffey disease [MIM: 114000]; Ehlers-Danlos syndrome 1 and 7a [MIM: 130000, 130060]
65-PRE	*COL1A2*	c.1378G>A (p.G460S) [NM_000089]	AD/de novo het	Recurrence risk	Up to 50%	RES	Osteogenesis imperfecta types 2–4 [MIM: 166210, 259420, 166220]; Ehlers-Danlos syndrome types 7B and cardiac valvular [MIM: 130060, 225320]
66-PRE	*COL1A2*	c.2576G>A (p.G859D) [NM_000089]	AD/de novo het	NR	NR	RES	Osteogenesis imperfecta types 2–4 [MIM: 166210, 259420, 166220]; Ehlers-Danlos syndrome types 7B and cardiac valvular [MIM: 130060, 225320]
53-P	*COL4A1*	c.2879G>T (p.G960V) [NM_001845]	AD/inherited het (mosaic mother)	Reproductive planning	NR	Up to 50%	Brain small vessel disease with hemorrhage [MIM: 607595]; Hereditary angiopathy with nephropathy aneurysms and muscle cramps [MIM: 611773]; Porencephaly 1 [MIM: 175780];
122-T	*DDX3X*	c.1703C>T (p.P568L) [NM_001193416]	XL, de novo het	NR	NR	RES	Mental retardation, X-linked 102 [MIM: 300958]
7-P	*DOK7*	c.437C>T (p.P146L) c.514G>A (p.G172R) [NM_173660]	AR, compound het	NR	NR	25%	Fetal akinesia deformation sequence [MIM: 208150]; Myasthenia, limb-girdle, familial [MIM: 254300]
101-PRE	*DVL1*	c.1519delT (p.W507fs) [NM_004421]	AD/de novo het	NR	NR	RES	Robinow syndrome, autosomal dominant 2 [MIM: 616331]
95-P	*DYNC2H1*	c.10885C>T (p.R3629X) c.11230C>T (p.L3744F) [NM_001080463]	AR/compound het	NR	NR	25%	Short-rib thoracic dysplasia 3 [MIM: 613091]
22-P	*EIF2B2*	c.586C>T (p.P196S) c.599G>T (p.G200V) [NM_014239]	AR/compound het	NR	NR	25%	Leukodystrophy with vanishing white matter [MIM: 603896]
81-PRE	*FBN1*	c.3299G>T (p.G1100V) [NM_000138]	AD/de novo het	NR	NR	RES	Marfan syndrome [MIM: 154700]; Geleophysic dysplasia 2 [MIM: 614185] MASS syndrome [MIM:604308]; Ectopia lentis, familial [MIM:129600]; Acromicric dysplasia [MIM:102370]; Marfan lipodystrophy syndrome [MIM: 616914]; Weill-Marchesani syndrome 2 [MIM: 608328]; Stiff skin syndrome [MIM:184900]
60-T	*FRMD4A*	c.2723C>T (p.S908L) [NM_018027]	AR/homozygous	NR	NR	25%	Agenesis of corpus callosum, with facial anomalies and cerebellar ataxia [MIM: 616819]
88-PRE	*GLI3*	c.3324C>G (p.Y1108X) [NM_000168]	AD/de novo het	NR	NR	RES	Pallister-Hall syndrome [MIM: 146510]; Greig cephalopolysyndactyly syndrome [MIM: 175700]; Polydactyly types A1 and B [MIM: 174200]; Polydactyly, type IV [MIM: 174700]
74-PRE	*HCCS*	c.308_309insAGT (p.V103dup) [NM_005333]	XL/de novo het	NR	NR	RES	Linear skin defects with multiple congenital anomalies [MIM: 309801]
114-T	*IFT80*	c.721G>C (p.G241R) [NM_020800]	AR/homozygous	Reproductive planning Recurrence risk	Unknown	25%	Short-rib thoracic dysplasia 2 with or without polydactyly [MIM: 611263]
96-P	*INTU*	c.1259+5G>T c.1714C>T (p.R572X) [NM_015693]	AR/compound het	NR	NR	25%	Ciliopathy with features of short-rib polydactyly syndrome
6-P	*KMT2D*	c.6617dupC (p.A2207fs) [NM_003482]	AD/de novo het	Reproductive planning Recurrence risk	Unknown	RES	Kabuki syndrome type 1 [MIM: 147920]
45-P	*KMT2D*	c.1967delT (p.L656fs) [NM_003482]	AD/ het (biological parents unavailable, gamete donors used)	Medical management Recurrence risk	Up to 25%	~ 0% (unless same donors used again)	Kabuki syndrome type 1 [MIM: 147920]
48-PRE	*KMT2D*	c.15680_15693dup (p.I5232fs) [NM_003482]	AD/de novo het	NR	NR	RES	Kabuki syndrome type 1 [MIM: 147920]
126-PRE	*KMT2D*	c.5707C>T (p.R1903X) [NM_003482]	AD/de novo het	NR	NR	RES	Kabuki syndrome type 1 [MIM: 147920]
55-PRE	*KRAS*	c.149C>T (p.T50I) [NM_004985]	AD/de novo het	NR	NR	RES	Noonan syndrome 3 [MIM: 609942]; Cardiofaciocutaneous syndrome [MIM: 115150]
63-PRE	*LAMC3*	c.4415G>A (p.R1472Q) c.4477+1G>A [NM_006059]	AR/compound het	NR	NR	25%	Cortical malformations occipital [MIM: 614115]
47-T	*MID1*	c.673_674delAG (p.S225X) [NM_000381]	XL/hemizygous (inherited from mildly affected mother)	NR	NR	50% (males)	Opitz GBBB syndrome 1 [MIM: 300000]
67-PRE	*MYH3*	c.2015G>A (p.R672H) [NM_002470]	AD/inherited het (mosaic mother)	NR	NR	Up to 50%	Arthrogryposis, distal types 2A, 2B, 8 [MIM: 193700, 601680, 178110]
20-P	*NDUFAF5*	c.29T>A (p.L10X) c.782T>G (p.M261R) [NM_024120]	AR/compound het	Reproductive planning Other (see Results)	25%	25%	Mitochondrial complex I deficiency [MIM: 252010]
1-P	*NIPBL*	c.459-2A>G [NM_133433]	AD/de novo het	Recurrence risk	Unknown	RES	Cornelia de Lange syndrome type 1 [MIM: 122470]
112-PRE	*P3H1*	c.12delC (p.R4fs) [NM_022356]	AR/homozygous	Reproductive planning Recurrence risk	25–50%	25%	Osteogenesis imperfecta 8 [MIM: 610915]
13-P	*PEX1*	c.2097dupT (p.I700fs) c.3205C>T (p.Q1069X) [NM_000466]	AR/compound het	Reproductive planning	NR	25%	Peroxisome biogenesis disorder types 1A, 1B [MIM: 214100, 601539]
46-PRE	*PKD1L1*	c.6473+2_6473+3del [NM_138295]	AR/homozygous	NR	NR	25%	Heterotaxy, visceral, 8, autosomal [MIM: 617205]
85-PRE	*PTPN11*	c.227A>T (p.E76V) [NM_002834]	AD/de novo mosaic	NR	NR	0%	Noonan syndrome 1 [MIM: 163950]; LEOPARD syndrome 1 [MIM: 151100]; Metachondromatosis [MIM: 156250];
144-PRE	*PTPN11*	c.854T>C (p.F285S) [NM_002834]	AD/de novo het	Reproductive planning	NR	RES	Noonan syndrome 1 [MIM: 163950]; LEOPARD syndrome 1 [MIM: 151100]; Metachondromatosis [MIM: 156250]
84-PRE	*RAPSN*	c.1166+1G>C [NM_005055]	AR/homozygous	NR	NR	25%	Fetal akinesia deformation sequence [MIM: 208150]; Myasthenic syndrome, congenital, 11 [MIM: 616326]
69-PRE	*RIT1*	c.246T>G (p.F82L) [NM_006912]	AD/de novo het	NR	NR	RES	Noonan syndrome 8 [MIM: 615355]
18-P	*RYR1*	c.14344G>A (p.G4782R) c.14512-1G>A [NM_000540]	AR/compound het	Medical management Reproductive planning	25%	25%	Central core disease of muscle [MIM: 117000]
133-PRE	*SOS1*	c.1655G>A (p.R552K) [NM_005633]	AD/de novo het	NR	NR	RES	Noonan Syndrome 4 [MIM: 610733]
11-P	*TMEM67*	c.1319G>A (p.R440Q) c.233G>A (p.C78Y) [NM_153704]	AR/compound het	Reproductive planning	25%	25%	Meckel syndrome 3 [MIM: 607361]; Joubert syndrome 6 [MIM: 610688]; Bardet-Biedl syndrome [MIM: 209900]; COACH syndrome [MIM: 216360]; Nephronophthisis 11 [MIM: 613550]
44-PRE	*TUBA1A*	c.1118G>A (p.R373K) [NM_006009]	AD/de novo het	Reproductive planning	Unknown	RES	Lissencephaly type 3 [MIM: 611603]
21-P	*WDR19*	c.275T>G (p.L92X) c.880G>A (p.G294R) [NM_025132]	AR/compound het	Medical management Reproductive planning Recurrence risk	Up to 25%	25%	Short-rib thoracic dysplasia 5 [MIM: 614376]; Cranioectodermal dysplasia 4 [MIM: 614378]; Nephronophthisis 13 [MIM: 614377]

Genes, variants, and diseases that contributed to the 46 molecular diagnoses from fetal exome sequencing. Case IDs ending in *-PRE* are prenatal trio exomes, those ending in *-T* are standard trio exomes, and those ending in *-P* are proband exomes

Abbreviations: *AD* autosomal dominant, *AR* autosomal recessive, *XL* X-linked, *het* heterozygous, *hom* homozygous, *hemi* hemizygous, *RES* residual recurrence risk due to possibility of parental germline mosaicism, *NR* information not received from ordering physicians

In the absence of professional practice guidelines for reporting incidental findings from prenatal exome sequencing, we defined an internal policy for such findings specifically for prenatal trio exomes (i.e., ongoing pregnancies). As described above (Methods), we included pathogenic and likely pathogenic variants in disease genes that are expected to cause significant childhood-onset disorders on the fetal report, even when not related to the prenatal indications. Reporting of such incidental findings was decided on a case-by-case basis with input from the ordering physician when necessary, taking into account factors such as disease severity and age of onset. Such findings were reported for 3 of the 62 prenatal trio exome tests (see Additional file 1: Table S3).

### Results by clinical indication categories

**Fig. 1:**
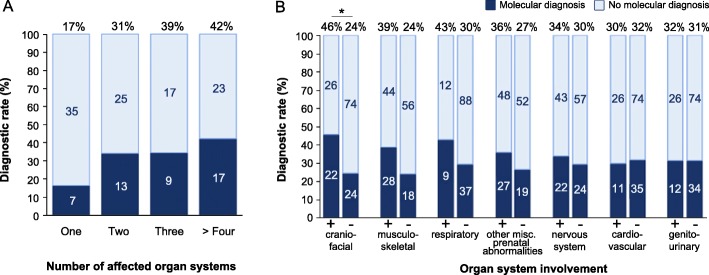
Molecular diagnostic rates based on phenotype. **a** Molecular diagnostic rate is higher in fetuses with abnormalities affecting multiple organ systems (*p* = 0.018; see Additional file 1: Table S5 for non-significant group comparisons). The number of fetuses in each category is indicated on the relevant bar graph. Each top-branch category was only counted once per fetus. **b** Molecular diagnostic rates are shown for fetuses with (+) or without (−) abnormalities in the stated organ system or top-level HPO category. Fetuses with craniofacial abnormalities were significantly more likely to receive a molecular diagnosis than those without (*p* = 0.013). Significant *p* values (*p* < 0.05) are indicated by (*), Fisher’s exact test

For all samples, the indication was one or more fetal abnormalities detected by prenatal imaging or autopsy. Clinical features that were provided by the referring physician(s) were converted into HPO terms using Phenomizer [27, 28] and grouped into top-branch HPO categories for each fetus (see Additional file 1: Table S4 for reported phenotypes and their corresponding categories). The number of unique top-branch HPO categories was tallied for each fetus. If a fetus had multiple abnormalities within the same top-branch category, that category was only counted once for that fetus. This analysis revealed that the molecular diagnostic rate in fetuses with abnormalities affecting multiple organ systems is higher compared to fetuses with abnormalities in a single organ system (*p* = 0.018, Fig. 1a and Additional file 1: Table S5). We next investigated whether the diagnostic rate was affected by the nature of the prenatal phenotype (Fig. 1b). Fetuses with craniofacial abnormalities had the highest diagnostic rate (46%, *n* = 22/48), and this was significantly higher than the rate among fetuses without such abnormalities (24%, *n* = 24/98, *p* = 0.013). Nearly half of all fetuses referred for exome sequencing (*n* = 72/146) had abnormalities affecting the muscular and/or skeletal system. The diagnostic rate for this group was 39% (*n* = 28/72), while 24% of fetuses without musculoskeletal abnormalities received a molecular diagnosis (*n* = 18/74, *p* = 0.075). Although only 14% of fetuses (*n* = 21/146) had abnormalities involving the respiratory system, this group had a diagnostic rate of 43% (*n* = 9/21). However, comparison to the diagnostic rate among fetuses without these abnormalities revealed no statistical difference (30%, *n* = 37/125, *p* = 0.309). Additional phenotypes that were frequently observed in this cohort affected the nervous system (*n* = 64/146), the cardiovascular system (*n* = 37/146), the genitourinary system (*n* = 38/146), and miscellaneous abnormalities specific to prenatal development (*n* = 75/146). The diagnostic rates in these groups ranged from 30 to 36%, which is similar to the overall diagnostic rate. Furthermore, the diagnostic rate did not significantly differ between fetuses with these respective abnormalities versus those without (*p* > 0.05). Abnormalities affecting the abdomen, spleen, thymus, and eye were rarely reported (< 10% of cases, Additional file 1: Table S4).

### Mendelian inheritance and role of family history

Among the 46 total molecular diagnoses, autosomal dominant (AD) disorders accounted for 50% (*n* = 23/46), autosomal recessive (AR) for 41% (*n* = 19/46), and 9% were due to X-linked (XL) disorders (*n* = 4/46; Table 3). The majority of the autosomal dominant disorders were caused by de novo variants (87%, *n* = 20/23; Table 3). Mosaicism of the contributing variant was detected in one of these fetuses (Case 85-PRE, Table 2). Inheritance of the variant from a mosaic mother was seen in two cases (Cases 53-P, 67-PRE), and parental samples were not available for one case (Case 45-PRE, Table 2). The majority of the AR disorders were due to compound heterozygous variants (68%, *n* = 13/19; Table 3). The remaining six AR diagnoses had homozygous contributing variants (32%, *n* = 6/19; Table 3). Four out of the six cases were determined to be the product of a consanguineous union based on family history and/or AOH data (Case 24-P, 60-T, 84-PRE, 112-PRE; Additional file 1: Table S6). Among the contributing variants in the four XL cases, two were de novo and heterozygous in female fetuses (Case 74-PRE and 122-T) and two were maternally inherited and hemizygous in male fetuses (Cases 111-T and 47-T, Tables 2 and 3).

**Table 3:** Inheritance pattern of genes and variants that contributed to molecular diagnoses

	All cases (*n* = 146 samples)	Sporadic (*n* = 106 samples)	Significant history (*n* = 40 samples)
Autosomal dominant (AD)			
De novo/germline	19	19	0
De novo/mosaic in fetus	1	1	0
Inherited/mosaic in mother	2	2	0
Parents unavailable	1	1	0
Total AD	23	23	0
Autosomal recessive (AR)			
Compound heterozygous	13	6	7
Homozygous	6	3	3
TOTAL AR	19	9	10
X-linked (XL)			
De novo	2	2	0
Inherited/mother	2	1	1
Total XL	4	3	1
Total molecular diagnoses	46	35	11

Cases classified as “sporadic” are those with no reported family members or previous pregnancies with a similar phenotype. Cases classified as “significant history” are those with a previous pregnancy or a close biological relative or with similar phenotypic findings

The diagnostic rate was not different in sporadic cases compared to those with a clinically ascertained significant family history (Table 3). Sporadic cases were defined as those in which the referred proband (fetus) was the first individual in the family to present with the specific phenotype, while cases were considered to have significant family history if a previous fetus or close biological relative had similar clinical features. The majority of cases were sporadic (73%, *n* = 106/146, Table 3). A diagnosis was made in 33% of sporadic cases (*n* = 35/106, Table 3). The majority of these were de novo variants associated with either AD (*n* = 20) or XL (*n* = 2) disorders (63%, *n* = 22/35, Table 3). These are associated with a much-reduced recurrence risk (RR), derived from the low likelihood of undetectable somatic or gonadal mosaicism in a parent. The remaining 11 cases had findings that indicated a higher RR of 25% due to homozygous or compound heterozygous variants associated with AR disorders (26%, *n* = 9/35), up to 50% due to an AD variant inherited from a mosaic parent (6%, *n* = 2/35), and 50% in males due to a maternally inherited XL variant (3%, *n* = 1/35; Table 3). Among 40 cases with a significant family history, 11 molecular diagnoses were made (28%, Table 3). All but one of these had biallelic variants associated with AR disorders, indicating a 25% RR (Table 3). The remaining case had a maternally inherited XL hemizygous variant, indicating a 50% RR in males (Table 3).

### Genes underlying frequent fetal diagnoses and novel fetal phenotypes of known disease genes

A frameshift (*n* = 3) or stopgain (*n* = 1) variant in an internal exon in *KMT2D* was reported for four fetuses (Case 6-P, 45-P, 48-PRE and 126-PRE, Table 2). These are all predicted to introduce a premature translation termination codon with non-sense mediated decay [29] resulting in a loss-of-function allele, making Kabuki syndrome, caused by haploinsufficiency of *KMT2D*, the most frequent single-gene disorder in this cohort [30]. In older children, Kabuki syndrome can be clinically diagnosed based on cardinal manifestations including characteristic facial features, abnormal limb/extremity features, microcephaly, short stature, and heart and kidney problems [31] that are neither apparent nor readily recognizable in neonates and infants [32, 33] and are even more challenging prenatally. Comparison of the phenotypes of the four fetuses with a molecular diagnosis of Kabuki syndrome suggests that the co-occurrence of complex cardiac defects (100%, *n* = 4/4) and renal structural anomalies (75%, *n* = 3/4) is a common prenatal presentation of this syndrome, which is consistent with described neonatal phenotypes of *KMT2D*-related Kabuki syndrome [19, 32]. Pathogenic missense variants in *COL1A1* or *COL1A2* were diagnosed in five fetuses (Case 17-P, 49-T, 90-PRE, 65-PRE, 66-PRE, Table 2) with a skeletal dysplasia phenotype, including shortened long bones and/or abnormalities of the thorax (*n* = 5), abnormalities of the skull (*n* = 2), absent fetal nasal bone (*n* = 1), edema (*n* = 1), intrauterine growth retardation (*n* = 1), abnormality of the umbilical cord (*n* = 1), cardiac abnormalities (*n* = 1), and genital abnormalities (*n* = 1) (Table 2). Notably, de novo variants in the *DDX3X* gene were reported for two female fetuses with cystic hygroma and edema (Case 37-PRE, Additional file 1: Table S3; 122-T, Table 2). Pathogenic variants in *DDX3X* are known to cause X-linked mental retardation disorder 102 (MRX102, MIM: 300958) in females and rarely in males [34], but have not previously been reported prenatally. The first identified de novo likely pathogenic variant was therefore initially reported as an incidental finding (Case 37-PRE, Additional file 1: Table S3), but the second, in a fetus with identical phenotype, was a known pathogenic variant and reported as a primary finding (Case 122-T, Table 2). One case (101-PRE) carried a de novo frameshift variant in *DVL1* previously reported in multiple patients with Robinow syndrome (DRS2, MIM: 616331). This variant is predicted to produce a premature termination codon in the last exon of *DVL1* and has been previously described to perturb Wnt signaling through a gain of function or dominant-negative mechanism [35–37]. The phenotype of this fetus, absent cavum pellucidum, abnormalities of the genitourinary system, skeletal system, head and neck, and suspected cardiac abnormality is consistent with that of individuals with *DVL1* pathogenic variants [35, 36].

### Clinical implications of receiving a molecular diagnosis

Information about the clinical implications of receiving an exome diagnosis was available for 14 of the 46 molecular diagnoses and scored as (1) altered medical management, (2) altered reproductive planning, (3) modified recurrence risk estimates, and (4) other impacts. In four cases, medical management was altered either by altering neonatal care or by informing pregnancy termination decisions (Table 2). For example, prenatal detection of a pathogenic *COL1A1* variant in case 90-PRE facilitated coordinating an appropriate perinatal care plan and connecting the parents with other families with osteogenesis imperfecta (OI) so they could learn practical skills for caring for a baby with OI. Recurrence risk estimates were modified or refined based on the molecular diagnosis in eight cases (Table 2), with a reported positive psychosocial impact for one family with a history of two previous deceased but undiagnosed infants (Table 2, Other, Case 20-P). Altered reproductive planning for future pregnancies, including targeted prenatal genetic testing or pre-implantation genetic diagnosis, was the most frequent clinical implication (*n* = 15 cases, Table 2). We are aware of at least 10 cases out of the total 46 molecular diagnoses where the WES result led to targeted testing in a future pregnancy. Additional feedback regarding pregnancy outcomes was provided to the lab for seven local cases (Additional file 1: Table S7).

## Discussion

The diagnostic yield of 32% in this cohort of 146 proband and trio exome sequencing tests performed on fetal samples is slightly higher than that of some recent larger series with reported diagnostic rates of 20–24% [38–40]. The subset of 62 ongoing pregnancies, with a diagnostic rate of 35%, is one of the first larger series reported to date where exome sequencing was done on still ongoing pregnancies. A prior review of studies, published and presented at international meetings with more than five cases each (range 7–101), indicated a diagnostic yield between 6 and 80% [20, 41–49]. This wide range is likely due to a combination of small sample sizes, differences in the a priori likelihood of an underlying Mendelian genetic etiology due to varying inclusion criteria, and variation in interpretation of pathogenicity between reports. Not surprisingly, our diagnostic yield of 32–35% is very close to recently reported 36.7% diagnostic yield from exome sequencing for 278 neonates and infants in intensive care units [19]. Some outcomes of exome sequencing on medical management that were described in this report will likely be applicable to prenatal exome sequencing [19], but more extensive and detailed prenatal studies will be required to further discern its clinical utility and refine clinical inclusion criteria for this advanced testing. We found a higher diagnostic rate for fetuses with structural abnormalities of multiple organ systems and for fetuses with abnormalities of craniofacial morphology, as well as a good diagnostic yield for prenatally detected musculoskeletal, respiratory, nervous system, cardiovascular, and genitourinary anomalies, suggesting clinicians may expect a higher yield in these prenatal presentations, after karyotype studies and chromosome microarray analysis are unrevealing. We further detected multiple large regions of AOH (> 5 Mb) on concurrent SNP array analysis in four fetal samples with homozygous variants [50]. These cases underscore that AOH, particularly as a result of consanguinity, can contribute to autosomal recessive disorders and influence the molecular diagnostic rate [45, 47, 51, 52]. While self-reported family history and SNP arrays provided adequate information to identify a molecular diagnosis for these samples, an alternative approach that could potentially improve sensitivity and reduce cost would be to test for AOH, CNVs, and uniparental disomy simultaneously by calculating the B allele frequency of all single nucleotide variants within the existing exome sequencing data [52].

Reporting VUS and incidental findings in prenatal exome results present a particular challenge because they can create a dilemma for clinicians, genetic counselors, and families who are considering difficult decisions for their pregnancy, delivery and neonatal management in a time-sensitive environment, which must be weighed against the risk of missing a potential molecular diagnosis if a VUS is not reported. Accurate variant interpretation and decisions whether to report a VUS can be compromised by incomplete communication between clinicians and the laboratory about the fetal phenotypic information. Another challenge is that current practice guidelines for reporting incidental findings from diagnostic exomes specifically excludes the prenatal setting [23, 24], although a very recent position statement has begun to address this [21]. To standardize our approach, we defined internal policies for prenatal exomes to report mainly pathogenic or likely pathogenic variants in genes related to prenatal testing indications or known to cause significant disorders during childhood, even if unrelated to the referring indications. We occasionally reported VUS on a case-by-case basis after multidisciplinary consensus decision between the laboratory and the ordering physician when there was a strong indication based on factors such as the presence of a pathogenic variant on the other allele in recessive disorders, good candidate gene based on the fetal phenotype, disease severity, and age of onset.

In the prenatal setting, the timeline for receiving diagnostic testing information is critical as couples may use the test results to support decisions for their pregnancy, including pregnancy continuation or termination, fetal treatment, and delivery management, as well as neonatal treatment. As turnaround time continues to decrease, the diagnostic results of prenatal exome sequencing will increasingly contribute to this decision-making process, in addition to its utility for recurrence risk counseling. We have demonstrated that initial exome results for ongoing pregnancies are routinely reported in ~ 2 weeks excluding cell culture time. Considering that in most cases exome sequencing is not initiated until the result of the CMA, which is usually performed in parallel to the cell culture on DNA directly extracted from the amniotic fluid sample, the need to wait for cell cultures often does not add to the overall time from procedure to the exome result (Additional file 1: Figure S2). Nevertheless, continued reduction in the time to molecular diagnosis remains possible. In addition to time required for specimen culturing, patients often wait for insurance verification, coverage determination, and cost estimates prior to initiating testing [53]. This impacts not only the turnaround time but also the emotional burden on the family.

Currently, little guidance on diagnostic prenatal exome sequencing exists. The ACMG currently recommends ES as an option for fetuses with multiple congenital anomalies suggestive of a genetic disorder for whom genetic tests that are specific to the phenotype have failed to determine a diagnosis [9]. Although the American College of Obstetricians and Gynecologists does not currently recommend the routine use of fetal exomes in the prenatal setting, they state that prenatal exome may be reasonable in select circumstances such as recurrent fetal phenotypes with no diagnosis by standard testing [4]. A recent joint position statement from the International Society for Prenatal Diagnosis, Society for Maternal and Fetal Medicine, and Perinatal Quality Foundation [21] further comments on reasonable indications for fetal exome testing and considers counseling and implementation aspects. Nevertheless, all current professional society statements emphasize the need for more peer-reviewed data regarding implementation of ES for prenatal diagnosis. Our study contributes such valuable information by reporting diagnostic rates, genotype-phenotype correlations, new information regarding prenatal presentations of some molecularly diagnosed disorders, and clinical impact of molecular diagnosis from a cohort of 146 consecutive fetal exomes sequenced and analyzed on a clinical basis.

## Conclusions

With rapid mean TAT of 2 weeks, we were able to provide molecular diagnosis for 35% of ongoing pregnancies that underwent prenatal trio exome analysis. An overall diagnostic rate of 32% was achieved including all sub-cohorts of proband, standard trio and prenatal trio exomes. We showed a higher molecular diagnostic rate in fetuses with structural anomalies in multiple organ systems and in fetuses with craniofacial abnormalities. Finally, we demonstrated that prenatal ES can offer substantial advantages for both families and clinicians, in terms of reproductive planning and decision-making, recurrence risk estimation, and medical management. Thus, our study demonstrates compelling evidence for the utility of prenatal exome sequencing as a promising new option in the realm of prenatal genetic diagnostics. We conclude that although more research on its clinical utility for various categories of fetal phenotypes is needed, prenatal exome sequencing can be offered in select cases, but should preferentially be implemented under guidance of experienced multidisciplinary teams that include prenatal genetics experts who work closely with laboratories experienced with both prenatal diagnosis and diagnostic genome-wide sequencing, as previously suggested [21].

## Additional file

Additional file 1: **Figure S1.** Comparison of proband versus trio exome workflows. **Figure S2.** Fetal sample types received and culturing time prior to prenatal exome sequencing. **Figure S3.** Referring practices. **Table S1.** Quality metrics of exome sequencing data of the fetal and parental samples. **Table S2.** Excluded samples without a final report. **Table S3.** Incidental findings reported for prenatal exome tests. **Table S4.** Reported fetal phenotypes. **Table S5.** Pairwise statistical analysis of diagnostic rate based on number of affected organ systems, corrected for multiple comparisons. **Table S6.** Regions of absence of heterozygosity (AOH) in cases with homozygous variants underlying the molecular diagnosis. **Table S7.** Pregnancy outcomes for locally referred cases. (PDF 8142 kb)

## Abbreviations

ACMG: American College of Medical Genetics and Genomics
AD: Autosomal dominant
AOH: Absence of heterozygosity
AR: Autosomal recessive
CAP: College of American Pathologists
CLIA: Clinical Laboratory Improvement Amendments
CMA: Chromosomal microarray analysis
CNV: Copy number variant
CVS: Chorionic villus samples
ES: Exome sequencing
het: Heterozygous
hom: Homozygous
hemi: Hemizygous
HPO: Human Phenotype Ontology
NGS: Next-generation sequencing
NR: Information not received from ordering physicians
OI: Osteogenesis imperfecta
POC: Product of conception
RES: Residual recurrence risk due to possibility of parental germline mosaicism
RR: Recurrence risk
SNP: Single nucleotide polymorphism
TAT: Turnaround time
VUS: Variant of unknown significance
XL: X-linked
