# Supplementary material for: Clinical exome sequencing for fetuses with ultrasound abnormalities and a suspected Mendelian disorder
Source: Genome Med. 2018 Sep 28;10:74. doi: 10.1186/s13073-018-0582-x (PMC6162951; doi:10.1186/s13073-018-0582-x)
Supplement: Supplementary file 1 — Figure S1. Comparison of proband versus trio exome workflows. Figure S2. Fetal sample types received and culturing time prior to prenatal exome sequencing. Figure S3. Referring practices. Table S1. Quality metrics of exome sequencing data of the fetal and parental samples. Table S2. Excluded samples without a final report. Table S3. Incidental findings reported for prenatal exome tests. Table S4. Reported fetal phenotypes. Table S5. Pairwise statistical analysis of diagnostic rate based on number of affected organ systems, corrected for multiple comparisons. Table S6. Regions of absence of heterozygosity (AOH) in cases with homozygous variants underlying the molecular diagnosis. Table S7. Pregnancy outcomes for locally referred cases. (PDF 8142 kb) [file 13073_2018_582_MOESM1_ESM.pdf]

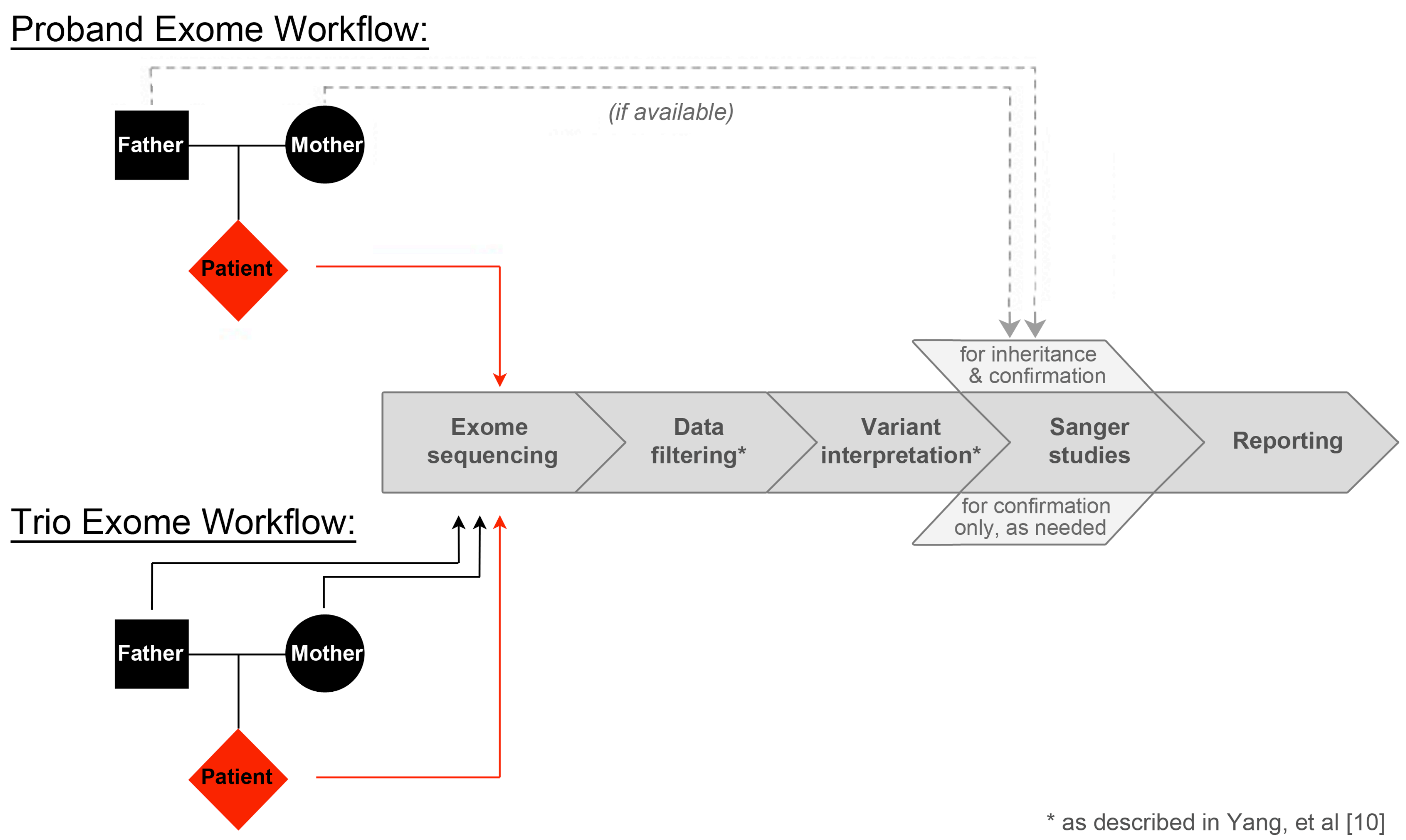


**Figure S1. Comparison of proband versus trio exome workflows**. Proband exome (top) requires only the patient sample to initiate exome sequencing. Sanger sequencing of clinically relevant variants is then performed on the patient and any available parental samples before final reporting. In contrast, trio exome tests (bottom) require both parental samples as well as the patient sample to initiate exome sequencing. For these tests, the patient and parental samples undergo exome sequencing simultaneously and results are interpreted in unison, taking into account de novo variants in the fetal DNA sample, inheritance and allelic configuration of each variant, as well as the clinical indications. Sanger studies are performed for confirmation only as necessary. The data filtering and variant interpretation steps are performed as previously described [10].

A

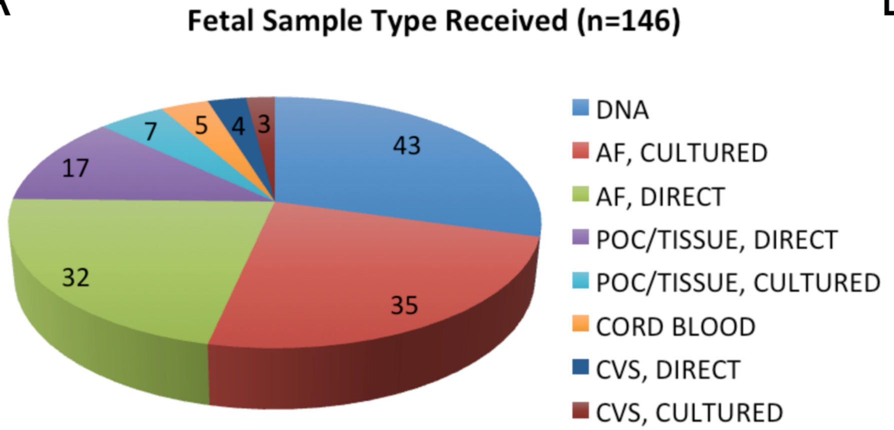


B

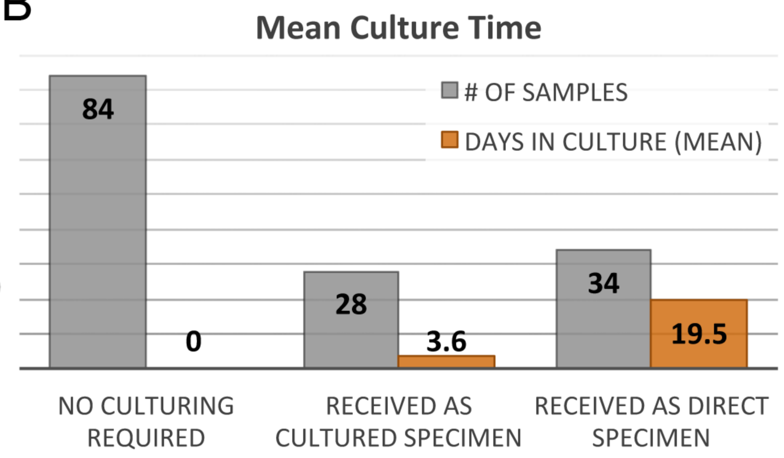


C

| Cohort | Sample state upon receipt | Timing of ES test order | Number of samples | Time in culture (days) mean | min | max | median |
|---|---|---|---|---|---|---|---|
| 1a | CULTURED | On day of sample receipt | 26 | 2.7 | 1 | 8 | 2 |
| 2a | CULTURED | After specimen was received - **total culture time** | 1 | 9 | 9 | 9 | 9 |
| | | After specimen was received – **culture time once ES order placed** | Same cohort as above (1) | 3 | 3 | 3 | 3 |
| 3a | CULTURED | After culturing and DNA extraction completed | 1 | 20 | 20 | 20 | 20 |
| 1b | DIRECT | On day of sample receipt | 7 | 16.4 | 12 | 21 | 17 |
| 2b | DIRECT | After specimen was received - **total culture time** | 21 | 19.7 | 12 | 44 | 17 |
| | | After specimen was received – **culture time once ES order placed** | Same cohort as above (21) | 6 | 1 | 14 | 6 |
| 3b | DIRECT | After culturing and DNA extraction completed | 6 | 22.5 | 14 | 33 | 21 |

**Figure S2. Fetal sample types received and culturing time prior to prenatal exome sequencing**. A. Sample types received for 146 proband, standard trio, and prenatal trio exome tests. B. 58% (n=84/146) of all fetal samples in this study did not require any culturing upon receipt, 19% (n=28/146) were received as cultured specimens but required an average of 3.6 days of additional culturing before DNA extraction, and 23% (n=34/146) were received as direct (uncultured) specimens and were cultured for 19.5 days on average. C. 18% (n=26/146) of cases were received as a cultured specimen with an ES order (Cohort 1a) but were determined to need additional culturing to bring sample confluency to sufficient levels for DNA extraction. Average culture time for Cohort 1a was 2.7 days. Only 5% of cases (n=7/146) were received as a direct specimen with an order for prenatal ES at the time of sample receipt (Cohort 1b; 6 AF, 1 CVS). The average culture time for Cohort 1b was 16.4 days. 47% (n=29/62) of the samples that required culturing after receipt did not arrive with an ES order (Cohorts 2a, 2b, 3a, 3b). Cohorts 2a and 2b are particularly complex to analyze because ES was ordered sometime after the original direct specimen was received. Both the total average culture time and the average culture time from receipt of ES order are listed for these cohorts. In 4% of cases (n=6/146), direct specimen was received, culture was grown, and DNA was extracted, at request of client with plans to add-on ES later (Cohort 3b). For these cases, culture time did not factor into total ES TAT, however average culture time was 22.5 days.  Abbreviations: AF, amniotic fluid; CVS, chorionic villi sample; POC, products of conception

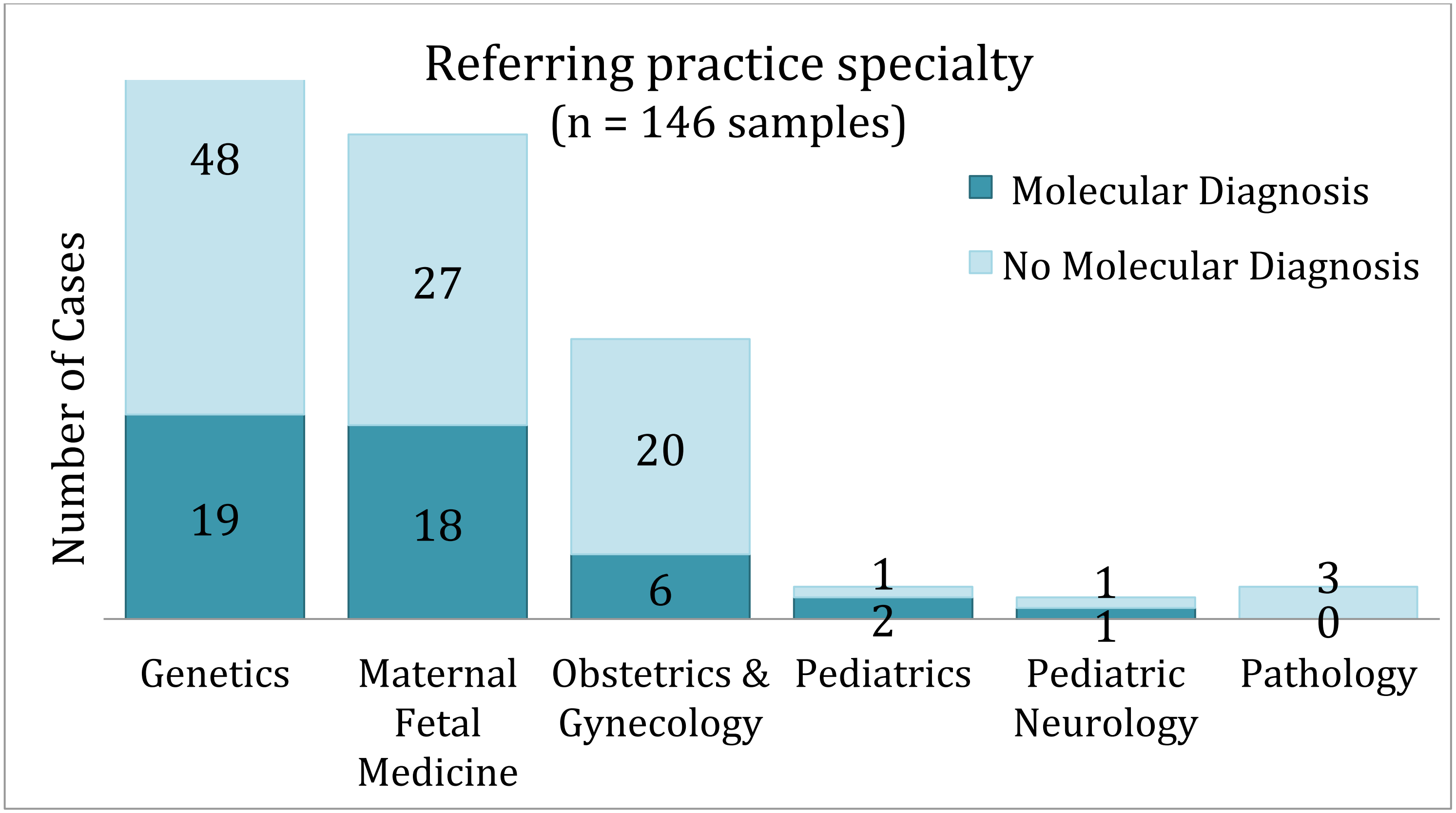


**Figure S3. Referring practices**. Number of cases with (dark) and without (light) a molecular diagnosis based on the specialty of the referring physician or practice. Genetics category includes: genetics, pediatric genetics, medical genetics, prenatal genetics, and reproductive genetics. Maternal fetal medicine category includes perinatology/neonatology.

**Table S1. Quality metrics of exome sequencing data of the fetal and parental samples.**

| Case ID [a] | Illumina Platform [b] | Unique Aligned (Mb) [c] | Total Pass filter (Mb) [d] | Avg % Align (PF) Read1 [e] | Avg % Align (PF) Read2 [f] | Avg % Error rate Read 1 [g] | Avg % Error rate Read 2 [h] | Unique-ness % [i] | Dupli-cate % [j] | Total reads aligned % [k] | Avg Coverage [l] | Reads Hit Target/ Buffer [m] | Bases 20+ Coverage [n] |
|---|---|---|---|---|---|---|---|---|---|---|---|---|---|
| 1 | HiSeq 2500 | 10876 | 11364 | 99.24 | 98.35 | 0.43 | 1.40 | 96.87 | 4.33 | 98.79 | 151 | 81 | 97.6 |
| 2 | HiSeq 2500 | 11863 | 12828 | 98.90 | 97.68 | 0.61 | 1.62 | 94.09 | 8.41 | 98.29 | 161 | 76 | 97.7 |
| 3 | HiSeq 2500 | 18348 | 20027 | 97.74 | 96.51 | 0.68 | 2.63 | 94.33 | 8.65 | 97.13 | 266 | 79 | 98.0 |
| 4 | HiSeq 2500 | 12633 | 13000 | 99.51 | 99.23 | 0.56 | 0.96 | 97.79 | 3.93 | 99.37 | 174 | 81 | 97.5 |
| 5 | HiSeq 2500 | 11730 | 12340 | 98.55 | 98.34 | 0.36 | 0.57 | 96.56 | 4.84 | 98.45 | 191 | 83 | 96.3 |
| 6 | HiSeq 2500 | 11200 | 11853 | 98.90 | 98.70 | 0.33 | 0.54 | 95.64 | 5.73 | 98.80 | 175 | 81 | 96.7 |
| 7 | HiSeq 2500 | 11696 | 12325 | 99.19 | 99.02 | 0.44 | 0.67 | 95.75 | 6.12 | 99.11 | 183 | 82 | 96.7 |
| 8 | HiSeq 2500 | 14042 | 15044 | 98.47 | 97.44 | 0.44 | 0.74 | 95.29 | 7.06 | 97.95 | 216 | 79 | 97.1 |
| 9 | HiSeq 2500 | 13533 | 14301 | 98.96 | 98.80 | 0.40 | 0.53 | 95.70 | 6.31 | 98.88 | 211 | 81 | 96.8 |
| 10 | HiSeq 2500 | 13015 | 13793 | 99.07 | 98.85 | 0.33 | 0.49 | 95.35 | 6.57 | 98.96 | 205 | 81 | 96.8 |
| 11 | HiSeq 2500 | 12408 | 13572 | 96.45 | 95.98 | 0.43 | 0.89 | 95.02 | 7.59 | 96.21 | 190 | 78 | 97.1 |
| 12 | HiSeq 2500 | 10941 | 11772 | 96.19 | 95.92 | 0.42 | 0.68 | 96.76 | 5.05 | 96.05 | 177 | 83 | 96.5 |
| 13 | HiSeq 2500 | 10359 | 10981 | 97.27 | 97.01 | 0.40 | 0.67 | 97.11 | 4.43 | 97.14 | 161 | 82 | 96.8 |
| 14 | HiSeq 2500 | 11458 | 12292 | 96.51 | 96.22 | 0.45 | 0.79 | 96.73 | 5.05 | 96.37 | 186 | 83 | 96.9 |
| 15 | HiSeq 2500 | 10978 | 11843 | 95.75 | 95.22 | 0.45 | 0.87 | 97.08 | 4.57 | 95.48 | 173 | 83 | 96.8 |
| 16 | HiSeq 2500 | 10017 | 11131 | 94.88 | 94.53 | 0.53 | 0.83 | 95.02 | 6.31 | 94.70 | 135 | 69 | 96.3 |
| 17 | HiSeq 2500 | 9490 | 9906 | 98.61 | 97.85 | 0.43 | 1.00 | 97.53 | 3.38 | 98.23 | 120 | 79 | 96.0 |
| 18 | HiSeq 2500 | 10636 | 11280 | 97.88 | 97.38 | 0.59 | 1.02 | 96.58 | 4.70 | 97.63 | 142 | 79 | 96.2 |
| 19 | HiSeq 2500 | 10582 | 11077 | 98.47 | 97.83 | 0.40 | 0.88 | 97.33 | 3.73 | 98.15 | 135 | 79 | 96.8 |
| 20 | HiSeq 2500 | 10771 | 11288 | 98.77 | 98.12 | 0.56 | 0.90 | 96.93 | 4.35 | 98.45 | 136 | 78 | 96.1 |
| 21 | HiSeq 2500 | 10985 | 11467 | 98.65 | 98.18 | 0.40 | 0.83 | 97.34 | 3.77 | 98.42 | 139 | 78 | 96.9 |
| 22 | HiSeq 2500 | 10697 | 11081 | 99.27 | 98.61 | 0.42 | 0.95 | 97.57 | 3.41 | 98.94 | 136 | 79 | 96.7 |
| 23 | HiSeq 2500 | 10639 | 11183 | 98.50 | 98.07 | 0.37 | 0.68 | 96.80 | 4.07 | 98.29 | 138 | 79 | 96.7 |
| 24 | HiSeq 2500 | 10426 | 10987 | 98.56 | 97.95 | 0.44 | 0.97 | 96.58 | 4.73 | 98.25 | 134 | 78 | 96.4 |
| 25 | HiSeq 2500 | 11140 | 11624 | 98.61 | 97.82 | 0.45 | 1.01 | 97.58 | 3.45 | 98.22 | 139 | 78 | 96.9 |
| 26 | HiSeq 2500 | 11416 | 11902 | 98.76 | 98.27 | 0.36 | 0.68 | 97.36 | 3.56 | 98.52 | 146 | 79 | 97.3 |
| 27 | HiSeq 2500 | 11985 | 12489 | 98.70 | 98.33 | 0.40 | 0.77 | 97.41 | 3.52 | 98.51 | 155 | 79 | 97.2 |
| 28 | HiSeq 2500 | 11627 | 12158 | 98.82 | 97.76 | 0.34 | 0.85 | 97.30 | 4.14 | 98.29 | 145 | 79 | 98.0 |
| 29 | HiSeq 2500 | 10456 | 10886 | 98.26 | 97.86 | 0.44 | 0.85 | 97.95 | 2.80 | 98.06 | 135 | 79 | 96.9 |
| 30 | HiSeq 2500 | 13406 | 13874 | 99.08 | 98.72 | 0.38 | 0.71 | 97.70 | 3.19 | 98.90 | 166 | 79 | 98.3 |

| | | | | | | | | | | | | | |
|---|---|---|---|---|---|---|---|---|---|---|---|---|---|
| 31 | HiSeq 2500 | 10855 | 11206 | 99.48 | 99.06 | 0.32 | 0.70 | 97.58 | 3.15 | 99.27 | 145 | 82 | 98.3 |
| 31-M | HiSeq 2500 | 12305 | 12726 | 99.44 | 99.13 | 0.30 | 0.59 | 97.39 | 3.35 | 99.29 | 161 | 82 | 98.2 |
| 31-P | HiSeq 2500 | 11073 | 11437 | 99.48 | 99.10 | 0.30 | 0.64 | 97.51 | 3.20 | 99.29 | 145 | 82 | 98.3 |
| 32 | HiSeq 2500 | 11381 | 11768 | 98.98 | 98.49 | 0.36 | 0.63 | 97.95 | 2.71 | 98.74 | 142 | 80 | 98.2 |
| 32-M | HiSeq 2500 | 11946 | 12328 | 99.06 | 98.63 | 0.35 | 0.62 | 98.03 | 2.70 | 98.84 | 151 | 80 | 98.2 |
| 32-P | HiSeq 2500 | 10167 | 10456 | 99.18 | 98.60 | 0.35 | 0.70 | 98.33 | 2.31 | 98.89 | 125 | 79 | 98.1 |
| 33 | HiSeq 2500 | 11005 | 11428 | 98.94 | 98.51 | 0.35 | 0.73 | 97.54 | 3.24 | 98.73 | 139 | 80 | 98.0 |
| 34 | HiSeq 2500 | 10893 | 11600 | 99.36 | 98.59 | 0.32 | 0.64 | 94.88 | 6.35 | 98.98 | 141 | 78 | 98.2 |
| 35 | HiSeq 2500 | 13230 | 13826 | 99.07 | 97.98 | 0.33 | 0.79 | 97.12 | 4.69 | 98.52 | 165 | 78 | 98.4 |
| 36 | HiSeq 2500 | 10670 | 11109 | 98.59 | 97.55 | 0.38 | 0.72 | 97.94 | 2.95 | 98.07 | 139 | 80 | 98.1 |
| 36-M | HiSeq 2500 | 12042 | 12574 | 98.52 | 97.45 | 0.37 | 0.70 | 97.74 | 3.22 | 97.98 | 155 | 80 | 98.3 |
| 36-P | HiSeq 2500 | 11732 | 12235 | 98.64 | 97.51 | 0.37 | 0.73 | 97.77 | 3.17 | 98.08 | 150 | 79 | 98.4 |
| 37 | HiSeq 2500 | 8802 | 9264 | 99.51 | 98.77 | 0.33 | 0.90 | 95.84 | 5.41 | 99.14 | 124 | 83 | 98.0 |
| 37-M | HiSeq 2500 | 13927 | 14538 | 99.43 | 98.82 | 0.33 | 0.77 | 96.64 | 4.48 | 99.13 | 175 | 78 | 98.3 |
| 37-P | HiSeq 2500 | 10822 | 11210 | 99.41 | 98.74 | 0.34 | 0.90 | 97.44 | 3.49 | 99.08 | 134 | 79 | 98.2 |
| 38 | HiSeq 2500 | 11143 | 11842 | 99.06 | 98.59 | 0.38 | 0.76 | 95.22 | 7.04 | 98.82 | 146 | 78 | 98.3 |
| 39 | HiSeq 2500 | 10080 | 10790 | 99.52 | 99.01 | 0.30 | 0.65 | 94.11 | 7.19 | 99.27 | 138 | 80 | 98.3 |
| 39-M | HiSeq 2500 | 10599 | 11261 | 99.43 | 99.12 | 0.29 | 0.56 | 94.81 | 6.30 | 99.28 | 142 | 79 | 98.2 |
| 39-P | HiSeq 2500 | 10000 | 10628 | 99.19 | 98.91 | 0.31 | 0.58 | 94.99 | 6.04 | 99.05 | 124 | 74 | 98.2 |
| 40 | HiSeq 2500 | 11743 | 12699 | 98.93 | 98.41 | 0.35 | 0.65 | 93.72 | 7.65 | 98.67 | 167 | 80 | 98.5 |
| 40-M | HiSeq 2500 | 10793 | 11584 | 98.62 | 98.02 | 0.38 | 0.76 | 94.76 | 6.54 | 98.32 | 141 | 74 | 98.1 |
| 40-P | HiSeq 2500 | 11449 | 12391 | 98.88 | 98.29 | 0.35 | 0.71 | 93.72 | 7.69 | 98.59 | 157 | 78 | 98.4 |
| 41 | HiSeq 2500 | 9849 | 10267 | 98.48 | 98.15 | 0.33 | 0.60 | 97.57 | 3.22 | 98.31 | 130 | 81 | 98.1 |
| 42 | HiSeq 2500 | 10781 | 12133 | 96.97 | 96.22 | 0.43 | 0.85 | 91.99 | 10.05 | 96.59 | 144 | 75 | 98.1 |
| 43 | HiSeq 2500 | 10797 | 11729 | 98.73 | 98.24 | 0.39 | 0.76 | 93.47 | 7.82 | 98.49 | 128 | 68 | 98.0 |
| 43-M | HiSeq 2500 | 10488 | 11385 | 98.81 | 98.36 | 0.35 | 0.69 | 93.44 | 7.94 | 98.58 | 144 | 78 | 98.2 |
| 43-P | HiSeq 2500 | 11422 | 12702 | 97.63 | 97.09 | 0.41 | 0.79 | 92.36 | 9.45 | 97.36 | 155 | 76 | 98.4 |
| 44 | HiSeq 2500 | 10610 | 11789 | 98.28 | 97.95 | 0.37 | 0.61 | 91.73 | 9.76 | 98.11 | 145 | 76 | 98.3 |
| 44-M | HiSeq 2500 | 11881 | 13187 | 98.39 | 98.11 | 0.37 | 0.62 | 91.70 | 9.83 | 98.25 | 159 | 75 | 98.3 |
| 44-P | HiSeq 2500 | 11021 | 12171 | 98.54 | 98.14 | 0.35 | 0.65 | 92.08 | 9.38 | 98.34 | 145 | 74 | 98.4 |
| 45 | HiSeq 2500 | 8594 | 9179 | 98.79 | 98.60 | 0.36 | 0.53 | 94.87 | 6.17 | 98.69 | 117 | 80 | 97.5 |
| 46 | HiSeq 2500 | 10650 | 11988 | 95.91 | 95.23 | 0.54 | 0.90 | 92.96 | 9.31 | 95.57 | 137 | 74 | 98.3 |
| 46-M | HiSeq 2500 | 12068 | 13351 | 98.69 | 98.15 | 0.41 | 0.74 | 91.84 | 10.14 | 98.42 | 164 | 77 | 98.3 |
| 46-P | HiSeq 2500 | 11431 | 12862 | 95.88 | 95.10 | 0.53 | 0.92 | 93.07 | 9.21 | 95.49 | 144 | 73 | 98.5 |
| 47 | HiSeq 2500 | 11547 | 12574 | 97.65 | 97.16 | 0.37 | 0.64 | 94.28 | 6.91 | 97.40 | 157 | 78 | 98.4 |
| 47-M | HiSeq 2500 | 10913 | 12082 | 96.53 | 95.94 | 0.40 | 0.71 | 93.86 | 7.65 | 96.24 | 144 | 76 | 98.2 |

| | | | | | | | | | | | | | |
|---|---|---|---|---|---|---|---|---|---|---|---|---|---|
| 47-P | HiSeq 2500 | 9505 | 10673 | 95.37 | 94.78 | 0.46 | 0.80 | 93.67 | 8.02 | 95.07 | 119 | 73 | 98.1 |
| 48 | HiSeq 2500 | 10110 | 11780 | 94.39 | 94.07 | 0.51 | 0.67 | 91.08 | 10.71 | 94.23 | 127 | 69 | 98.1 |
| 48-M | HiSeq 2500 | 10258 | 11919 | 94.53 | 94.23 | 0.49 | 0.67 | 91.19 | 10.58 | 94.38 | 127 | 69 | 98.0 |
| 48-P | HiSeq 2500 | 10375 | 12152 | 93.89 | 93.52 | 0.53 | 0.71 | 91.11 | 10.86 | 93.70 | 130 | 69 | 98.1 |
| 49 | HiSeq 2500 | 11407 | 12870 | 99.09 | 98.66 | 0.42 | 0.68 | 89.64 | 12.80 | 98.87 | 158 | 75 | 98.5 |
| 49-M | HiSeq 2500 | 9721 | 10649 | 99.21 | 98.95 | 0.26 | 0.51 | 92.13 | 9.04 | 99.08 | 135 | 78 | 98.1 |
| 49-P | HiSeq 2500 | 11268 | 12430 | 99.26 | 98.65 | 0.26 | 0.55 | 91.61 | 9.70 | 98.95 | 150 | 76 | 98.6 |
| 50 | HiSeq 2500 | 10812 | 12273 | 96.33 | 95.97 | 0.46 | 0.67 | 91.62 | 9.51 | 96.15 | 110 | 56 | 97.2 |
| 50-M | HiSeq 2500 | 10752 | 12132 | 97.03 | 96.82 | 0.41 | 0.57 | 91.44 | 9.57 | 96.92 | 108 | 55 | 97.2 |
| 50-P | HiSeq 2500 | 10769 | 11404 | 99.48 | 99.27 | 0.32 | 0.56 | 95.03 | 6.01 | 99.37 | 143 | 80 | 98.2 |
| 51 | HiSeq 2500 | 10000 | 11336 | 95.23 | 94.87 | 0.55 | 0.76 | 92.81 | 8.99 | 95.05 | 129 | 72 | 97.9 |
| 51-M | HiSeq 2500 | 10361 | 11829 | 94.53 | 94.08 | 0.58 | 0.81 | 92.88 | 9.14 | 94.30 | 130 | 70 | 97.9 |
| 51-P | HiSeq 2500 | 11599 | 13532 | 92.43 | 91.82 | 0.62 | 0.95 | 93.04 | 9.29 | 92.13 | 133 | 67 | 98.4 |
| 52 | HiSeq 2500 | 10191 | 11636 | 96.45 | 96.21 | 0.43 | 0.60 | 90.92 | 10.60 | 96.33 | 134 | 72 | 98.1 |
| 52-M | HiSeq 2500 | 12083 | 13869 | 96.29 | 95.93 | 0.44 | 0.65 | 90.65 | 11.24 | 96.11 | 155 | 71 | 98.3 |
| 52-P | HiSeq 2500 | 9416 | 10829 | 95.61 | 95.13 | 0.46 | 0.71 | 91.17 | 10.55 | 95.37 | 119 | 70 | 98.0 |
| 53 | HiSeq 2500 | 12466 | 14586 | 94.47 | 93.29 | 0.52 | 0.93 | 91.04 | 12.64 | 93.88 | 148 | 66 | 98.5 |
| 54 | HiSeq 2500 | 10097 | 11659 | 97.68 | 97.15 | 0.38 | 0.57 | 88.90 | 12.59 | 97.42 | 114 | 60 | 97.6 |
| 55 | HiSeq 2500 | 10214 | 12181 | 92.81 | 91.99 | 0.53 | 0.80 | 90.75 | 11.44 | 92.40 | 125 | 67 | 97.7 |
| 55-M | HiSeq 2500 | 10043 | 11946 | 92.98 | 92.33 | 0.51 | 0.77 | 90.73 | 11.41 | 92.65 | 121 | 66 | 97.7 |
| 55-P | HiSeq 2500 | 10824 | 12927 | 92.83 | 91.97 | 0.57 | 0.89 | 90.62 | 12.10 | 92.40 | 126 | 64 | 98.0 |
| 56 | HiSeq 2500 | 9030 | 10098 | 96.37 | 95.96 | 0.40 | 0.61 | 92.99 | 8.47 | 96.16 | 121 | 75 | 97.8 |
| 56-M | HiSeq 2500 | 10027 | 11270 | 95.92 | 95.40 | 0.42 | 0.68 | 93.01 | 8.68 | 95.66 | 129 | 73 | 97.9 |
| 56-P | HiSeq 2500 | 9532 | 10728 | 95.49 | 94.93 | 0.43 | 0.72 | 93.32 | 8.31 | 95.21 | 120 | 72 | 97.9 |
| 57 | HiSeq 2500 | 12158 | 13474 | 97.39 | 96.75 | 0.35 | 0.65 | 92.96 | 8.80 | 97.07 | 166 | 76 | 98.5 |
| 57-M | HiSeq 2500 | 13124 | 14516 | 97.70 | 97.09 | 0.34 | 0.65 | 92.83 | 8.99 | 97.40 | 179 | 77 | 98.4 |
| 57-P | HiSeq 2500 | 11766 | 12994 | 97.59 | 96.87 | 0.34 | 0.71 | 93.13 | 8.63 | 97.23 | 160 | 77 | 98.5 |
| 58 | HiSeq 2500 | 12722 | 13546 | 98.47 | 97.09 | 0.36 | 0.90 | 96.05 | 5.64 | 97.78 | 167 | 80 | 98.4 |
| 59 | HiSeq 2500 | 9715 | 10858 | 98.96 | 97.61 | 0.34 | 0.83 | 91.03 | 10.99 | 98.29 | 129 | 73 | 98.0 |
| 59-M | HiSeq 2500 | 8365 | 9182 | 98.97 | 97.55 | 0.29 | 0.84 | 92.71 | 8.92 | 98.26 | 109 | 74 | 97.4 |
| 59-P | HiSeq 2500 | 11511 | 12850 | 99.03 | 97.64 | 0.31 | 0.81 | 91.10 | 10.99 | 98.33 | 150 | 72 | 98.5 |
| 60 | HiSeq 2500 | 9225 | 9766 | 98.38 | 97.88 | 0.31 | 0.60 | 96.26 | 5.23 | 98.13 | 118 | 78 | 97.2 |
| 60-M | HiSeq 2500 | 13573 | 14235 | 98.61 | 98.31 | 0.31 | 0.58 | 96.84 | 4.08 | 98.46 | 173 | 79 | 98.3 |
| 60-P | HiSeq 2500 | 13073 | 13743 | 98.43 | 97.81 | 0.31 | 0.70 | 96.95 | 4.06 | 98.12 | 164 | 78 | 98.4 |
| 61 | HiSeq 2500 | 10228 | 10876 | 98.72 | 97.89 | 0.33 | 0.74 | 95.66 | 5.46 | 98.31 | 133 | 79 | 97.7 |
| 62 | HiSeq 2500 | 10757 | 12607 | 92.84 | 91.91 | 0.60 | 0.90 | 92.37 | 10.57 | 92.38 | 128 | 66 | 98.1 |

| | | | | | | | | | | | | | |
|---|---|---|---|---|---|---|---|---|---|---|---|---|---|
| 62-M | HiSeq 2500 | 10355 | 12073 | 93.06 | 91.99 | 0.57 | 0.90 | 92.70 | 10.15 | 92.53 | 119 | 66 | 97.7 |
| 62-P | HiSeq 2500 | 9787 | 11452 | 92.72 | 91.58 | 0.57 | 0.90 | 92.74 | 10.12 | 92.15 | 114 | 66 | 97.7 |
| 63 | HiSeq 2500 | 12552 | 13189 | 98.83 | 96.49 | 0.35 | 2.37 | 97.45 | 5.45 | 97.66 | 158 | 79 | 98.1 |
| 63-M | HiSeq 2500 | 12725 | 13342 | 98.99 | 96.75 | 0.33 | 2.35 | 97.45 | 5.50 | 97.87 | 156 | 78 | 98.1 |
| 63-P | HiSeq 2500 | 13533 | 14194 | 98.84 | 96.36 | 0.33 | 2.40 | 97.69 | 5.14 | 97.60 | 161 | 76 | 98.4 |
| 64 | HiSeq 2500 | 10938 | 12170 | 99.22 | 98.60 | 0.26 | 0.46 | 90.87 | 10.45 | 98.91 | 145 | 74 | 98.4 |
| 65 | HiSeq 2500 | 11188 | 12222 | 98.94 | 97.45 | 0.31 | 0.88 | 93.22 | 8.58 | 98.19 | 143 | 73 | 98.2 |
| 65-M | HiSeq 2500 | 12490 | 13622 | 98.98 | 97.86 | 0.31 | 0.83 | 93.16 | 8.63 | 98.42 | 159 | 73 | 98.4 |
| 65-P | HiSeq 2500 | 11720 | 12797 | 98.91 | 97.77 | 0.31 | 0.84 | 93.13 | 8.67 | 98.34 | 148 | 73 | 98.4 |
| 66 | HiSeq 2500 | 11831 | 12804 | 99.27 | 98.89 | 0.29 | 0.51 | 93.26 | 7.85 | 99.08 | 159 | 77 | 98.4 |
| 66-M | HiSeq 2500 | 10255 | 11010 | 99.25 | 98.70 | 0.28 | 0.69 | 94.11 | 7.00 | 98.98 | 131 | 75 | 98.1 |
| 66-P | HiSeq 2500 | 11836 | 12827 | 99.24 | 98.99 | 0.29 | 0.50 | 93.10 | 8.08 | 99.11 | 157 | 76 | 98.5 |
| 67 | HiSeq 2500 | 10168 | 11559 | 99.11 | 98.42 | 0.34 | 0.70 | 89.07 | 13.09 | 98.77 | 147 | 76 | 98.1 |
| 67-M | HiSeq 2500 | 11457 | 13006 | 99.29 | 98.49 | 0.28 | 0.68 | 89.08 | 13.53 | 98.89 | 153 | 74 | 98.4 |
| 67-P | HiSeq 2500 | 11539 | 13141 | 99.24 | 98.55 | 0.28 | 0.63 | 88.79 | 13.76 | 98.90 | 155 | 74 | 98.5 |
| 68 | HiSeq 2500 | 12873 | 14047 | 98.62 | 97.68 | 0.30 | 0.57 | 93.37 | 8.04 | 98.15 | 171 | 78 | 98.2 |
| 68-M | HiSeq 2500 | 12833 | 14020 | 98.64 | 97.78 | 0.29 | 0.55 | 93.20 | 8.28 | 98.21 | 167 | 77 | 98.2 |
| 68-P | HiSeq 2500 | 12994 | 14113 | 98.75 | 97.86 | 0.29 | 0.59 | 93.66 | 7.75 | 98.31 | 163 | 76 | 98.5 |
| 69 | HiSeq 2500 | 9861 | 10865 | 98.57 | 97.61 | 0.34 | 0.64 | 92.53 | 9.27 | 98.09 | 133 | 76 | 98.2 |
| 69-M | HiSeq 2500 | 11047 | 12087 | 98.76 | 97.83 | 0.32 | 0.63 | 92.98 | 8.71 | 98.30 | 149 | 76 | 98.3 |
| 69-P | HiSeq 2500 | 10872 | 11932 | 98.60 | 97.75 | 0.32 | 0.61 | 92.81 | 8.88 | 98.17 | 145 | 76 | 98.4 |
| 70 | HiSeq 2500 | 9814 | 11172 | 98.92 | 98.62 | 0.29 | 0.52 | 88.94 | 13.34 | 98.77 | 131 | 72 | 98.0 |
| 70-M | HiSeq 2500 | 9740 | 10955 | 98.82 | 98.40 | 0.30 | 0.60 | 90.16 | 11.43 | 98.61 | 133 | 74 | 98.0 |
| 70-P | HiSeq 2500 | 10478 | 11799 | 98.78 | 98.52 | 0.30 | 0.48 | 90.02 | 11.37 | 98.65 | 143 | 74 | 98.4 |
| 71 | HiSeq 2500 | 11920 | 13384 | 99.34 | 97.79 | 0.27 | 0.67 | 90.36 | 11.84 | 98.56 | 166 | 76 | 98.5 |
| 71-M | HiSeq 2500 | 11387 | 12769 | 99.39 | 98.28 | 0.26 | 0.62 | 90.23 | 11.85 | 98.84 | 159 | 77 | 98.3 |
| 71-P | HiSeq 2500 | 12045 | 13479 | 99.37 | 98.24 | 0.27 | 0.68 | 90.44 | 11.73 | 98.81 | 168 | 77 | 98.6 |
| 72 | HiSeq 2500 | 11015 | 12411 | 99.07 | 97.90 | 0.33 | 0.62 | 90.12 | 12.24 | 98.49 | 145 | 72 | 98.2 |
| 72-M | HiSeq 2500 | 11325 | 12718 | 99.10 | 97.87 | 0.32 | 0.67 | 90.42 | 12.00 | 98.48 | 145 | 71 | 98.2 |
| 72-P | HiSeq 2500 | 11446 | 12868 | 99.06 | 97.73 | 0.33 | 0.72 | 90.40 | 12.10 | 98.39 | 145 | 71 | 98.5 |
| 73 | HiSeq 2500 | 10058 | 10829 | 99.24 | 98.65 | 0.29 | 0.59 | 93.87 | 7.41 | 98.94 | 131 | 78 | 97.7 |
| 73-M | HiSeq 2500 | 11293 | 12163 | 99.31 | 98.74 | 0.28 | 0.59 | 93.76 | 7.57 | 99.02 | 146 | 77 | 98.1 |
| 73-P | HiSeq 2500 | 10944 | 11815 | 99.27 | 98.59 | 0.29 | 0.68 | 93.63 | 7.97 | 98.93 | 142 | 77 | 98.3 |
| 74 | HiSeq 2500 | 9450 | 11018 | 98.77 | 97.74 | 0.32 | 0.51 | 87.29 | 14.80 | 98.25 | 133 | 72 | 97.7 |
| 74-M | HiSeq 2500 | 9235 | 10578 | 98.73 | 97.56 | 0.31 | 0.58 | 88.95 | 13.05 | 98.15 | 128 | 73 | 97.7 |
| 74-P | HiSeq 2500 | 11546 | 13471 | 98.77 | 97.56 | 0.31 | 0.56 | 87.31 | 15.01 | 98.17 | 160 | 71 | 98.4 |

| | | | | | | | | | | | | | |
|---|---|---|---|---|---|---|---|---|---|---|---|---|---|
| 75 | HiSeq 2500 | 9263 | 10119 | 98.38 | 98.12 | 0.31 | 0.46 | 93.17 | 8.04 | 98.25 | 120 | 76 | 97.1 |
| 75-M | HiSeq 2500 | 11423 | 12359 | 98.87 | 98.64 | 0.30 | 0.47 | 93.59 | 7.40 | 98.76 | 143 | 75 | 97.8 |
| 75-P | HiSeq 2500 | 9873 | 10666 | 98.91 | 98.65 | 0.30 | 0.50 | 93.71 | 7.23 | 98.78 | 120 | 74 | 97.4 |
| 76 | HiSeq 2500 | 10996 | 12073 | 99.51 | 99.07 | 0.24 | 0.45 | 91.73 | 9.38 | 99.29 | 153 | 78 | 98.5 |
| 76-M | HiSeq 2500 | 10325 | 11307 | 99.57 | 99.17 | 0.23 | 0.44 | 91.89 | 9.16 | 99.37 | 142 | 78 | 98.2 |
| 76-P | HiSeq 2500 | 12684 | 13913 | 99.55 | 99.13 | 0.24 | 0.46 | 91.77 | 9.41 | 99.34 | 173 | 77 | 98.7 |
| 77 | HiSeq 2500 | 11287 | 12035 | 98.91 | 98.45 | 0.31 | 0.52 | 95.04 | 5.94 | 98.68 | 150 | 79 | 98.0 |
| 77-M | HiSeq 2500 | 10750 | 11446 | 98.96 | 98.51 | 0.30 | 0.52 | 95.12 | 5.95 | 98.73 | 141 | 79 | 97.9 |
| 77-P | HiSeq 2500 | 10463 | 11150 | 98.98 | 98.59 | 0.30 | 0.50 | 94.99 | 6.07 | 98.79 | 136 | 79 | 98.0 |
| 78 | HiSeq 2500 | 10670 | 11757 | 99.38 | 99.08 | 0.26 | 0.46 | 91.46 | 9.90 | 99.23 | 148 | 77 | 98.1 |
| 78-M | HiSeq 2500 | 11189 | 12344 | 99.42 | 99.09 | 0.25 | 0.47 | 91.32 | 10.12 | 99.25 | 154 | 77 | 98.3 |
| 78-P | HiSeq 2500 | 9580 | 10568 | 99.39 | 99.00 | 0.26 | 0.52 | 91.39 | 10.15 | 99.20 | 131 | 77 | 98.1 |
| 79 | HiSeq 2500 | 10598 | 11396 | 98.58 | 97.60 | 0.33 | 0.74 | 94.81 | 6.71 | 98.09 | 137 | 77 | 97.8 |
| 79-M | HiSeq 2500 | 13403 | 14391 | 98.86 | 97.82 | 0.32 | 0.75 | 94.71 | 6.94 | 98.34 | 169 | 76 | 98.1 |
| 79-P | HiSeq 2500 | 10463 | 11210 | 98.74 | 97.72 | 0.32 | 0.78 | 95.02 | 6.45 | 98.23 | 131 | 76 | 97.7 |
| 80 | HiSeq 2500 | 12506 | 13650 | 97.51 | 96.89 | 0.61 | 0.80 | 94.26 | 7.46 | 97.20 | 150 | 71 | 98.0 |
| 80-M | HiSeq 2500 | 9301 | 10092 | 97.98 | 97.26 | 0.59 | 0.82 | 94.41 | 7.29 | 97.62 | 106 | 70 | 96.1 |
| 80-P | HiSeq 2500 | 11697 | 12666 | 98.23 | 97.48 | 0.58 | 0.82 | 94.37 | 7.33 | 97.86 | 136 | 70 | 98.0 |
| 81 | HiSeq 2500 | 10909 | 12122 | 98.78 | 97.65 | 0.27 | 0.48 | 91.63 | 10.04 | 98.21 | 152 | 77 | 98.2 |
| 81-M | HiSeq 2500 | 10768 | 12001 | 98.58 | 97.42 | 0.28 | 0.52 | 91.56 | 10.13 | 98.00 | 150 | 77 | 98.2 |
| 81-P | HiSeq 2500 | 10771 | 11937 | 98.81 | 97.64 | 0.28 | 0.52 | 91.86 | 9.75 | 98.22 | 149 | 77 | 98.4 |
| 82 | HiSeq 2500 | 11054 | 12598 | 98.67 | 97.87 | 0.30 | 0.56 | 89.29 | 12.54 | 98.27 | 158 | 77 | 98.4 |
| 82-M | HiSeq 2500 | 12075 | 13693 | 98.99 | 98.14 | 0.28 | 0.55 | 89.47 | 12.34 | 98.57 | 171 | 76 | 98.4 |
| 82-P | HiSeq 2500 | 12444 | 14078 | 99.11 | 98.13 | 0.28 | 0.60 | 89.63 | 12.24 | 98.62 | 172 | 75 | 98.6 |
| 83 | HiSeq 2500 | 12644 | 14088 | 99.36 | 98.72 | 0.28 | 0.64 | 90.62 | 11.30 | 99.04 | 177 | 77 | 98.6 |
| 83-M | HiSeq 2500 | 11637 | 12915 | 99.37 | 98.75 | 0.26 | 0.63 | 90.96 | 10.88 | 99.06 | 160 | 77 | 98.3 |
| 83-P | HiSeq 2500 | 12242 | 13630 | 99.35 | 98.76 | 0.27 | 0.61 | 90.67 | 11.20 | 99.06 | 170 | 77 | 98.6 |
| 84 | HiSeq 2500 | 11315 | 11966 | 99.28 | 98.69 | 0.28 | 0.79 | 95.53 | 5.64 | 98.99 | 149 | 80 | 98.2 |
| 84-M | HiSeq 2500 | 11666 | 12351 | 99.29 | 98.44 | 0.28 | 0.84 | 95.54 | 5.68 | 98.86 | 154 | 80 | 98.1 |
| 84-P | HiSeq 2500 | 10866 | 11481 | 99.27 | 98.53 | 0.28 | 0.91 | 95.70 | 5.46 | 98.90 | 142 | 80 | 98.1 |
| 85 | HiSeq 2500 | 9770 | 11231 | 99.41 | 98.72 | 0.25 | 0.51 | 87.81 | 13.82 | 99.06 | 140 | 76 | 98.0 |
| 85-M | HiSeq 2500 | 11056 | 12723 | 99.39 | 98.51 | 0.24 | 0.57 | 87.82 | 14.00 | 98.95 | 154 | 74 | 98.3 |
| 85-P | HiSeq 2500 | 8927 | 10289 | 99.40 | 98.71 | 0.25 | 0.50 | 87.59 | 14.11 | 99.06 | 126 | 75 | 97.9 |
| 86 | HiSeq 2500 | 11410 | 13007 | 99.41 | 99.16 | 0.28 | 0.45 | 88.35 | 13.34 | 99.29 | 162 | 76 | 98.4 |
| 86-M | HiSeq 2500 | 10941 | 12473 | 99.33 | 98.99 | 0.27 | 0.48 | 88.46 | 13.24 | 99.16 | 151 | 75 | 98.3 |
| 86-P | HiSeq 2500 | 9773 | 11107 | 99.38 | 99.06 | 0.27 | 0.47 | 88.68 | 12.91 | 99.22 | 135 | 75 | 98.3 |

| | | | | | | | | | | | | | |
|---|---|---|---|---|---|---|---|---|---|---|---|---|---|
| 87 | HiSeq 2500 | 9978 | 11559 | 99.40 | 99.00 | 0.25 | 0.49 | 87.02 | 14.88 | 99.20 | 139 | 74 | 98.1 |
| 87-M | HiSeq 2500 | 10659 | 12425 | 99.30 | 98.84 | 0.26 | 0.48 | 86.59 | 15.44 | 99.07 | 150 | 73 | 98.2 |
| 87-P | HiSeq 2500 | 11074 | 12853 | 99.39 | 99.01 | 0.26 | 0.47 | 86.85 | 15.02 | 99.20 | 155 | 74 | 98.5 |
| 88 | HiSeq 2500 | 10249 | 11496 | 99.20 | 98.70 | 0.28 | 0.54 | 90.10 | 11.71 | 98.95 | 140 | 75 | 98.1 |
| 88-M | HiSeq 2500 | 11163 | 12505 | 99.19 | 98.81 | 0.28 | 0.52 | 90.17 | 11.60 | 99.00 | 153 | 75 | 98.3 |
| 88-P | HiSeq 2500 | 9913 | 11085 | 99.17 | 98.73 | 0.28 | 0.55 | 90.38 | 11.36 | 98.95 | 134 | 75 | 98.2 |
| 89 | HiSeq 2500 | 12730 | 14331 | 99.46 | 98.47 | 0.27 | 0.55 | 89.76 | 12.07 | 98.96 | 182 | 77 | 98.5 |
| 89-M | HiSeq 2500 | 11854 | 13370 | 99.50 | 98.54 | 0.26 | 0.57 | 89.54 | 12.44 | 99.02 | 167 | 76 | 98.5 |
| 89-P | HiSeq 2500 | 10969 | 12323 | 99.43 | 98.42 | 0.26 | 0.62 | 89.98 | 11.86 | 98.92 | 155 | 77 | 98.5 |
| 90 | HiSeq 2500 | 11402 | 12982 | 99.31 | 98.14 | 0.28 | 0.66 | 88.96 | 13.05 | 98.72 | 159 | 74 | 98.4 |
| 90-M | HiSeq 2500 | 11199 | 12745 | 99.32 | 98.36 | 0.28 | 0.63 | 88.90 | 13.13 | 98.84 | 153 | 74 | 98.2 |
| 90-P | HiSeq 2500 | 10281 | 11603 | 99.31 | 97.95 | 0.28 | 0.91 | 89.84 | 12.30 | 98.63 | 133 | 72 | 98.3 |
| 91 | HiSeq 2500 | 11089 | 11790 | 98.40 | 98.06 | 0.33 | 0.64 | 95.75 | 5.33 | 98.23 | 147 | 79 | 97.8 |
| 91-M | HiSeq 2500 | 9286 | 9847 | 98.52 | 98.23 | 0.32 | 0.60 | 95.86 | 5.11 | 98.37 | 125 | 80 | 97.5 |
| 91-P | HiSeq 2500 | 11227 | 11915 | 98.57 | 98.19 | 0.33 | 0.65 | 95.78 | 5.25 | 98.38 | 146 | 79 | 98.1 |
| 92 | HiSeq 2500 | 12932 | 13694 | 99.51 | 98.68 | 0.26 | 0.61 | 95.30 | 5.97 | 99.10 | 169 | 79 | 98.2 |
| 92-M | HiSeq 2500 | 13061 | 13807 | 99.51 | 98.70 | 0.25 | 0.65 | 95.45 | 5.76 | 99.10 | 167 | 78 | 98.3 |
| 92-P | HiSeq 2500 | 12070 | 12769 | 99.48 | 98.60 | 0.25 | 0.69 | 95.44 | 5.83 | 99.04 | 154 | 78 | 98.3 |
| 93 | HiSeq 2500 | 12908 | 13708 | 99.35 | 98.46 | 0.30 | 0.67 | 95.21 | 6.43 | 98.91 | 167 | 79 | 98.2 |
| 93-M | HiSeq 2500 | 13045 | 13833 | 99.27 | 98.39 | 0.30 | 0.70 | 95.42 | 6.15 | 98.83 | 169 | 79 | 98.2 |
| 93-P | HiSeq 2500 | 11418 | 12081 | 99.31 | 98.49 | 0.31 | 0.68 | 95.56 | 5.90 | 98.90 | 148 | 79 | 98.1 |
| 94 | HiSeq 2500 | 8886 | 10082 | 98.96 | 98.08 | 0.33 | 0.66 | 89.46 | 12.44 | 98.52 | 114 | 69 | 97.4 |
| 94-M | HiSeq 2500 | 10686 | 12056 | 99.14 | 98.12 | 0.30 | 0.66 | 89.87 | 12.01 | 98.63 | 135 | 69 | 98.0 |
| 94-P | HiSeq 2500 | 12172 | 13753 | 98.98 | 98.03 | 0.31 | 0.64 | 89.85 | 12.11 | 98.51 | 153 | 69 | 98.5 |
| 95 | HiSeq 2500 | 10998 | 11872 | 98.88 | 98.54 | 0.29 | 0.56 | 93.85 | 7.36 | 98.71 | 147 | 78 | 97.9 |
| 96 | HiSeq 2500 | 11161 | 12010 | 98.42 | 97.70 | 0.33 | 0.54 | 94.77 | 6.61 | 98.06 | 150 | 79 | 97.9 |
| 97 | HiSeq 2500 | 10059 | 12612 | 98.79 | 98.27 | 0.31 | 0.67 | 80.95 | 24.52 | 98.53 | 138 | 66 | 98.4 |
| 97-M | HiSeq 2500 | 11830 | 13748 | 99.32 | 98.85 | 0.27 | 0.60 | 86.84 | 15.30 | 99.08 | 167 | 74 | 98.4 |
| 97-P | HiSeq 2500 | 11955 | 13866 | 99.27 | 98.84 | 0.27 | 0.61 | 87.04 | 15.06 | 99.06 | 169 | 74 | 98.6 |
| 98 | HiSeq 2500 | 11122 | 12537 | 98.78 | 98.38 | 0.32 | 0.58 | 89.99 | 11.65 | 98.58 | 152 | 74 | 98.2 |
| 98-M | HiSeq 2500 | 10066 | 11373 | 98.70 | 98.41 | 0.31 | 0.60 | 89.81 | 11.88 | 98.55 | 136 | 73 | 98.0 |
| 98-P | HiSeq 2500 | 10072 | 11365 | 98.64 | 98.30 | 0.33 | 0.61 | 90.00 | 11.70 | 98.47 | 136 | 73 | 98.2 |
| 99 | HiSeq 2500 | 9298 | 10528 | 98.53 | 97.70 | 0.30 | 0.51 | 90.01 | 11.57 | 98.12 | 128 | 74 | 97.8 |
| 100 | HiSeq 2500 | 9457 | 10892 | 97.21 | 96.90 | 0.38 | 0.57 | 89.46 | 12.52 | 97.05 | 142 | 75 | 97.3 |
| 101 | HiSeq 2500 | 10940 | 12340 | 99.35 | 98.83 | 0.27 | 0.58 | 89.47 | 12.19 | 99.09 | 149 | 74 | 98.2 |
| 101-M | HiSeq 2500 | 10827 | 12323 | 99.19 | 98.76 | 0.28 | 0.57 | 88.77 | 13.25 | 98.98 | 149 | 73 | 98.2 |

| | | | | | | | | | | | | | |
|---|---|---|---|---|---|---|---|---|---|---|---|---|---|
| 101-P | HiSeq 2500 | 11604 | 13296 | 99.22 | 98.81 | 0.28 | 0.53 | 88.14 | 14.09 | 99.02 | 159 | 72 | 98.5 |
| 102 | HiSeq 2500 | 10471 | 11776 | 98.48 | 97.75 | 0.30 | 0.52 | 90.63 | 10.94 | 98.12 | 144 | 74 | 98.0 |
| 102-M | HiSeq 2500 | 12359 | 13861 | 98.72 | 97.96 | 0.29 | 0.53 | 90.67 | 11.00 | 98.34 | 169 | 74 | 98.3 |
| 102-P | HiSeq 2500 | 10933 | 12262 | 98.69 | 97.90 | 0.29 | 0.56 | 90.71 | 10.93 | 98.29 | 148 | 74 | 98.2 |
| 103 | HiSeq 2500 | 10139 | 11422 | 99.15 | 98.78 | 0.28 | 0.60 | 89.70 | 11.95 | 98.97 | 146 | 77 | 98.3 |
| 103-M | HiSeq 2500 | 11967 | 13449 | 99.23 | 98.90 | 0.27 | 0.57 | 89.82 | 11.86 | 99.07 | 172 | 77 | 98.4 |
| 103-P | HiSeq 2500 | 11295 | 12719 | 98.84 | 98.51 | 0.29 | 0.57 | 90.00 | 11.58 | 98.68 | 162 | 77 | 98.5 |
| 104 | HiSeq 2500 | 11828 | 12523 | 98.88 | 98.18 | 0.29 | 0.54 | 95.86 | 5.51 | 98.53 | 160 | 80 | 98.2 |
| 104-M | HiSeq 2500 | 11665 | 12237 | 99.12 | 98.34 | 0.26 | 0.61 | 96.55 | 4.57 | 98.73 | 154 | 80 | 98.0 |
| 104-P | HiSeq 2500 | 13186 | 13881 | 99.07 | 98.40 | 0.27 | 0.55 | 96.21 | 4.95 | 98.74 | 172 | 80 | 98.4 |
| 105 | HiSeq 2500 | 11785 | 12740 | 98.62 | 98.38 | 0.33 | 0.56 | 93.91 | 7.23 | 98.50 | 150 | 76 | 98.0 |
| 105-M | HiSeq 2500 | 12855 | 13894 | 98.84 | 98.62 | 0.30 | 0.54 | 93.71 | 7.49 | 98.73 | 166 | 76 | 98.0 |
| 105-P | HiSeq 2500 | 10574 | 11417 | 98.64 | 98.38 | 0.32 | 0.58 | 94.02 | 7.11 | 98.51 | 134 | 75 | 97.6 |
| 106 | HiSeq 2500 | 12174 | 12884 | 98.99 | 98.71 | 0.31 | 0.54 | 95.59 | 5.63 | 98.85 | 161 | 79 | 98.0 |
| 106-M | HiSeq 2500 | 12509 | 13123 | 99.11 | 98.85 | 0.30 | 0.60 | 96.30 | 4.64 | 98.98 | 165 | 80 | 98.1 |
| 106-P | HiSeq 2500 | 13457 | 14197 | 99.15 | 98.95 | 0.29 | 0.52 | 95.70 | 5.44 | 99.05 | 176 | 79 | 98.5 |
| 107 | HiSeq 2500 | 11297 | 13092 | 98.74 | 98.11 | 0.34 | 0.63 | 87.67 | 14.57 | 98.43 | 168 | 74 | 98.2 |
| 107-M | HiSeq 2500 | 13150 | 15273 | 99.04 | 98.26 | 0.29 | 0.64 | 87.28 | 15.48 | 98.65 | 180 | 71 | 98.4 |
| 107-P | HiSeq 2500 | 12893 | 14886 | 98.95 | 98.26 | 0.30 | 0.64 | 87.84 | 14.63 | 98.60 | 178 | 72 | 98.6 |
| 108 | HiSeq 2500 | 10490 | 12173 | 97.47 | 97.12 | 0.36 | 0.65 | 88.57 | 14.67 | 97.30 | 150 | 75 | 98.3 |
| 108-M | HiSeq 2500 | 11734 | 13052 | 99.44 | 99.17 | 0.28 | 0.52 | 90.53 | 11.18 | 99.30 | 165 | 78 | 98.4 |
| 108-P | HiSeq 2500 | 11834 | 13140 | 99.40 | 99.17 | 0.29 | 0.51 | 90.71 | 11.00 | 99.28 | 167 | 78 | 98.6 |
| 109 | HiSeq 2500 | 11335 | 12727 | 99.35 | 99.09 | 0.31 | 0.55 | 89.76 | 12.06 | 99.22 | 160 | 76 | 98.4 |
| 109-M | HiSeq 2500 | 11702 | 13119 | 99.17 | 98.88 | 0.31 | 0.56 | 90.08 | 11.63 | 99.03 | 166 | 76 | 98.3 |
| 109-P | HiSeq 2500 | 10340 | 11599 | 99.09 | 98.80 | 0.31 | 0.58 | 90.10 | 11.59 | 98.94 | 147 | 76 | 98.3 |
| 110 | HiSeq 2500 | 10877 | 12951 | 98.94 | 98.17 | 0.29 | 0.55 | 85.22 | 16.93 | 98.56 | 157 | 73 | 98.2 |
| 110-M | HiSeq 2500 | 10925 | 12742 | 99.10 | 98.27 | 0.27 | 0.62 | 86.88 | 15.33 | 98.68 | 156 | 74 | 98.2 |
| 110-P | HiSeq 2500 | 9844 | 11687 | 99.06 | 98.34 | 0.28 | 0.55 | 85.34 | 16.81 | 98.70 | 141 | 73 | 98.2 |
| 111 | HiSeq 2500 | 10231 | 11107 | 98.42 | 98.15 | 0.36 | 0.62 | 93.72 | 7.58 | 98.29 | 137 | 78 | 97.7 |
| 111-M | HiSeq 2500 | 11308 | 12250 | 98.48 | 98.22 | 0.35 | 0.64 | 93.86 | 7.44 | 98.35 | 150 | 78 | 97.9 |
| 111-P | HiSeq 2500 | 13594 | 14710 | 98.60 | 98.28 | 0.34 | 0.68 | 93.88 | 7.45 | 98.44 | 182 | 78 | 98.5 |
| 112 | HiSeq 2500 | 10165 | 13015 | 98.30 | 97.99 | 0.33 | 0.68 | 79.58 | 23.13 | 98.14 | 140 | 64 | 98.0 |
| 112-M | HiSeq 2500 | 12069 | 15331 | 98.64 | 98.37 | 0.31 | 0.66 | 79.92 | 22.76 | 98.51 | 164 | 64 | 98.3 |
| 112-P | HiSeq 2500 | 9593 | 13190 | 98.59 | 98.30 | 0.31 | 0.55 | 73.88 | 28.55 | 98.45 | 136 | 61 | 97.8 |
| 113 | HiSeq 2500 | 10688 | 11810 | 98.15 | 97.46 | 0.34 | 0.66 | 92.53 | 8.86 | 97.81 | 141 | 75 | 97.9 |
| 113-M | HiSeq 2500 | 10139 | 11114 | 98.09 | 97.32 | 0.33 | 0.74 | 93.37 | 7.99 | 97.70 | 135 | 76 | 97.5 |

| | | | | | | | | | | | | | |
|---|---|---|---|---|---|---|---|---|---|---|---|---|---|
| 113-P | HiSeq 2500 | 10180 | 11302 | 97.89 | 97.08 | 0.35 | 0.70 | 92.40 | 9.07 | 97.49 | 134 | 75 | 97.6 |
| 114 | HiSeq 2500 | 11430 | 13074 | 98.52 | 98.23 | 0.34 | 0.57 | 88.87 | 13.03 | 98.37 | 168 | 76 | 98.5 |
| 114-M | HiSeq 2500 | 12208 | 13797 | 98.70 | 98.33 | 0.31 | 0.63 | 89.82 | 12.16 | 98.52 | 173 | 77 | 98.4 |
| 114-P | HiSeq 2500 | 11272 | 12926 | 98.09 | 97.80 | 0.33 | 0.55 | 89.03 | 12.75 | 97.95 | 160 | 76 | 98.5 |
| 115 | HiSeq 2500 | 12320 | 14045 | 97.93 | 96.89 | 0.35 | 0.61 | 90.05 | 12.24 | 97.41 | 172 | 74 | 98.5 |
| 116 | HiSeq 2500 | 10411 | 11253 | 98.12 | 97.94 | 0.34 | 0.56 | 94.38 | 6.65 | 98.03 | 139 | 79 | 97.8 |
| 116-M | HiSeq 2500 | 10732 | 11619 | 97.96 | 97.82 | 0.34 | 0.56 | 94.36 | 6.71 | 97.89 | 145 | 79 | 97.8 |
| 116-P | HiSeq 2500 | 12422 | 13390 | 98.33 | 98.13 | 0.33 | 0.59 | 94.44 | 6.64 | 98.23 | 165 | 79 | 98.3 |
| 117 | HiSeq 2500 | 10472 | 11537 | 97.25 | 95.82 | 0.42 | 0.76 | 94.03 | 7.55 | 96.54 | 127 | 71 | 97.8 |
| 117-M | HiSeq 2500 | 11399 | 12486 | 97.72 | 97.43 | 0.34 | 0.58 | 93.56 | 7.60 | 97.57 | 153 | 78 | 97.9 |
| 117-P | HiSeq 2500 | 11172 | 12246 | 97.44 | 97.08 | 0.35 | 0.60 | 93.80 | 7.39 | 97.26 | 150 | 78 | 98.1 |
| 118 | HiSeq 2500 | 9849 | 11600 | 97.95 | 97.59 | 0.34 | 0.53 | 86.84 | 14.95 | 97.77 | 135 | 71 | 98.0 |
| 119 | HiSeq 2500 | 13152 | 15713 | 96.71 | 96.33 | 0.38 | 0.67 | 86.72 | 17.01 | 96.52 | 175 | 67 | 98.4 |
| 119-M | HiSeq 2500 | 9144 | 10455 | 98.16 | 97.85 | 0.34 | 0.63 | 89.24 | 12.57 | 98.00 | 125 | 72 | 97.3 |
| 119-P | HiSeq 2500 | 13771 | 15744 | 98.59 | 98.26 | 0.32 | 0.65 | 88.87 | 13.38 | 98.43 | 184 | 71 | 98.5 |
| 120 | HiSeq 2500 | 12031 | 13380 | 98.6 | 98.31 | 0.33 | 0.54 | 91.33 | 10.31 | 98.45 | 173 | 78 | 98.4 |
| 120-M | HiSeq 2500 | 10606 | 11792 | 98.30 | 98.08 | 0.34 | 0.54 | 91.60 | 9.86 | 98.19 | 148 | 76 | 98.1 |
| 120-P | HiSeq 2500 | 12422 | 13848 | 98.07 | 97.77 | 0.34 | 0.59 | 91.61 | 10.03 | 97.92 | 173 | 77 | 98.6 |
| 121 | HiSeq 2500 | 12442 | 14077 | 98.5 | 97.48 | 0.33 | 0.61 | 90.2 | 11.82 | 97.99 | 172 | 74 | 98.4 |
| 121-M | HiSeq 2500 | 10246 | 11626 | 97.92 | 95.70 | 0.36 | 0.69 | 91.03 | 11.46 | 96.81 | 124 | 66 | 97.6 |
| 121-P | HiSeq 2500 | 10080 | 11445 | 97.89 | 95.81 | 0.37 | 0.72 | 90.94 | 11.52 | 96.85 | 120 | 66 | 97.8 |
| 122 | HiSeq 2500 | 10942 | 12483 | 98.83 | 97.51 | 0.31 | 0.82 | 89.29 | 13.12 | 98.17 | 154 | 74 | 98.2 |
| 122-M | HiSeq 2500 | 11886 | 13752 | 97.80 | 96.99 | 0.34 | 0.64 | 88.74 | 13.80 | 97.40 | 167 | 73 | 98.4 |
| 122-P | HiSeq 2500 | 12418 | 14281 | 98.04 | 97.26 | 0.34 | 0.65 | 89.05 | 13.41 | 97.65 | 174 | 74 | 98.6 |
| 123 | HiSeq 2500 | 11605 | 12888 | 98.28 | 97.72 | 0.43 | 0.8 | 91.88 | 9.91 | 98.00 | 155 | 71 | 98.0 |
| 123-M | HiSeq 2500 | 10567 | 11548 | 99.25 | 98.74 | 0.31 | 0.66 | 92.43 | 9.26 | 98.99 | 149 | 78 | 98.1 |
| 123-P | HiSeq 2500 | 10223 | 11164 | 99.19 | 98.55 | 0.31 | 0.77 | 92.62 | 9.14 | 98.87 | 143 | 78 | 98.2 |
| 124 | HiSeq 2500 | 11660 | 14301 | 97.4 | 96.75 | 0.35 | 0.62 | 83.99 | 18.66 | 97.07 | 161 | 68 | 98.4 |
| 124-M | HiSeq 2500 | 8953 | 11180 | 96.25 | 95.72 | 0.35 | 0.57 | 83.43 | 19.30 | 95.98 | 127 | 69 | 97.4 |
| 124-P | HiSeq 2500 | 8097 | 10512 | 92.01 | 91.39 | 0.36 | 0.61 | 84.00 | 18.46 | 91.70 | 115 | 69 | 97.1 |
| 125 | HiSeq 2500 | 12267 | 13494 | 98.51 | 98.22 | 0.33 | 0.61 | 92.42 | 8.77 | 98.37 | 154 | 75 | 98.2 |
| 126 | HiSeq 2500 | 11146 | 12381 | 98.33 | 97.33 | 0.35 | 0.82 | 92.02 | 9.71 | 97.83 | 148 | 72 | 98.1 |
| 126-M | HiSeq 2500 | 11456 | 12961 | 97.49 | 96.81 | 0.38 | 0.70 | 90.98 | 11.21 | 97.15 | 152 | 71 | 98.1 |
| 126-P | HiSeq 2500 | 12715 | 14147 | 98.16 | 97.52 | 0.36 | 0.68 | 91.86 | 10.06 | 97.84 | 167 | 71 | 98.5 |
| 127 | HiSeq 2500 | 12550 | 13791 | 98.96 | 98.78 | 0.31 | 0.55 | 92.04 | 9.77 | 98.87 | 179 | 78 | 98.4 |
| 127-M | HiSeq 2500 | 11178 | 12240 | 98.69 | 98.36 | 0.33 | 0.64 | 92.69 | 9.10 | 98.52 | 162 | 79 | 98.2 |

| | | | | | | | | | | | | | |
|---|---|---|---|---|---|---|---|---|---|---|---|---|---|
| 127-P | HiSeq 2500 | 11855 | 13139 | 98.63 | 98.41 | 0.33 | 0.57 | 91.58 | 10.52 | 98.52 | 170 | 78 | 98.5 |
| 128 | HiSeq 2500 | 10078 | 10752 | 98.7 | 98.48 | 0.38 | 0.61 | 95.07 | 6.13 | 98.59 | 136 | 80 | 97.7 |
| 128-M | HiSeq 2500 | 8831 | 9800 | 98.70 | 98.55 | 0.30 | 0.44 | 91.37 | 9.93 | 98.62 | 121 | 75 | 97.3 |
| 128-P | HiSeq 2500 | 8685 | 9574 | 98.67 | 98.48 | 0.30 | 0.48 | 92.03 | 9.23 | 98.57 | 119 | 76 | 97.4 |
| 129 | HiSeq 2500 | 12758 | 13651 | 98.09 | 96.7 | 0.41 | 1.06 | 95.96 | 5.98 | 97.40 | 144 | 69 | 97.9 |
| 130 | HiSeq 2500 | 11374 | 12223 | 98.68 | 97.31 | 0.37 | 0.74 | 94.96 | 6.69 | 97.99 | 147 | 77 | 98.0 |
| 130-M | HiSeq 2500 | 12472 | 13395 | 98.84 | 97.55 | 0.34 | 0.74 | 94.82 | 6.87 | 98.20 | 159 | 77 | 98.0 |
| 130-P | HiSeq 2500 | 12129 | 13066 | 98.62 | 96.98 | 0.35 | 0.91 | 94.92 | 6.89 | 97.80 | 155 | 77 | 98.1 |
| 131 | HiSeq 2500 | 10916 | 11495 | 99.5 | 98.45 | 0.25 | 0.78 | 95.95 | 5.27 | 98.97 | 141 | 80 | 97.7 |
| 131-M | HiSeq 2500 | 13258 | 14044 | 99.55 | 98.45 | 0.24 | 0.89 | 95.36 | 6.41 | 99.00 | 171 | 79 | 98.1 |
| 131-P | HiSeq 2500 | 12081 | 12795 | 99.50 | 98.11 | 0.25 | 1.13 | 95.56 | 6.20 | 98.81 | 156 | 80 | 98.1 |
| 132 | HiSeq 2500 | 14336 | 15138 | 99.54 | 99.18 | 0.29 | 0.61 | 95.31 | 6.4 | 99.36 | 185 | 80 | 98.5 |
| 132-M | HiSeq 2500 | 12523 | 13184 | 99.54 | 99.03 | 0.28 | 0.73 | 95.67 | 6.10 | 99.29 | 160 | 80 | 98.0 |
| 132-P | HiSeq 2500 | 12374 | 13052 | 99.53 | 99.14 | 0.29 | 0.60 | 95.44 | 6.16 | 99.33 | 160 | 80 | 98.2 |
| 133 | HiSeq 2500 | 10376 | 11422 | 99.15 | 98.78 | 0.33 | 0.68 | 91.79 | 10.27 | 98.97 | 142 | 77 | 98.2 |
| 133-M | HiSeq 2500 | 11478 | 12639 | 99.16 | 98.84 | 0.34 | 0.63 | 91.73 | 10.30 | 99.00 | 162 | 77 | 98.2 |
| 133-P | HiSeq 2500 | 12186 | 13446 | 99.18 | 98.81 | 0.33 | 0.65 | 91.55 | 10.61 | 98.99 | 172 | 77 | 98.5 |
| 134 | HiSeq 2500 | 10374 | 11345 | 99.18 | 98.89 | 0.3 | 0.51 | 92.33 | 8.97 | 99.04 | 134 | 73 | 98.0 |
| 134-M | HiSeq 2500 | 11111 | 12150 | 99.16 | 98.86 | 0.31 | 0.50 | 92.36 | 8.88 | 99.01 | 145 | 73 | 98.0 |
| 134-P | HiSeq 2500 | 11189 | 12250 | 99.08 | 98.76 | 0.31 | 0.52 | 92.34 | 9.01 | 98.92 | 145 | 73 | 98.2 |
| 135 | HiSeq 2500 | 10432 | 12449 | 99.08 | 98.3 | 0.28 | 0.59 | 84.91 | 17.42 | 98.69 | 142 | 68 | 97.1 |
| 136 | HiSeq 2500 | 11406 | 13642 | 98.53 | 98.22 | 0.34 | 0.62 | 84.99 | 16.95 | 98.38 | 157 | 67 | 97.8 |
| 136-M | HiSeq 2500 | 11179 | 13117 | 98.59 | 98.12 | 0.33 | 0.78 | 86.65 | 15.42 | 98.35 | 149 | 68 | 97.7 |
| 136-P | HiSeq 2500 | 12826 | 15295 | 98.60 | 98.27 | 0.34 | 0.61 | 85.19 | 16.76 | 98.43 | 172 | 67 | 98.3 |
| 137 | HiSeq 2500 | 12987 | 13751 | 99.04 | 98.05 | 0.32 | 0.66 | 95.84 | 5.49 | 98.55 | 166 | 77 | 98.5 |
| 137-M | HiSeq 2500 | 12645 | 13388 | 99.12 | 98.14 | 0.31 | 0.67 | 95.76 | 5.60 | 98.63 | 161 | 77 | 98.1 |
| 137-P | HiSeq 2500 | 10092 | 10702 | 99.02 | 97.89 | 0.32 | 0.78 | 95.78 | 5.65 | 98.46 | 127 | 76 | 97.4 |
| 138 | HiSeq 2500 | 13646 | 14566 | 99.41 | 98.63 | 0.29 | 0.77 | 94.61 | 6.93 | 99.02 | 176 | 78 | 98.2 |
| 138-M | HiSeq 2500 | 13400 | 14342 | 99.40 | 98.64 | 0.28 | 0.77 | 94.36 | 7.27 | 99.02 | 174 | 78 | 98.1 |
| 138-P | HiSeq 2500 | 11766 | 12554 | 99.40 | 98.47 | 0.30 | 0.91 | 94.73 | 6.84 | 98.94 | 151 | 78 | 98.0 |
| 139 | HiSeq 2500 | 13797 | 14863 | 99.14 | 98.85 | 0.28 | 0.44 | 93.77 | 7.32 | 99.00 | 182 | 78 | 98.4 |
| 139-M | HiSeq 2500 | 10992 | 11762 | 99.06 | 98.76 | 0.27 | 0.47 | 94.48 | 6.53 | 98.91 | 147 | 79 | 97.6 |
| 139-P | HiSeq 2500 | 11075 | 11947 | 99.07 | 98.71 | 0.29 | 0.47 | 93.74 | 7.38 | 98.89 | 146 | 78 | 97.8 |
| 140 | HiSeq 2500 | 8916 | 10783 | 98.76 | 97.78 | 0.33 | 0.81 | 84.14 | 18.73 | 98.27 | 125 | 68 | 96.8 |
| 140-M | HiSeq 2500 | 12328 | 14836 | 98.92 | 98.20 | 0.29 | 0.62 | 84.31 | 18.21 | 98.56 | 171 | 68 | 98.2 |
| 140-P | HiSeq 2500 | 9978 | 11951 | 98.99 | 98.00 | 0.29 | 0.81 | 84.77 | 17.95 | 98.49 | 136 | 68 | 97.7 |

| | | | | | | | | | | | | | |
|---|---|---|---|---|---|---|---|---|---|---|---|---|---|
| 141 | HiSeq 2500 | 11203 | 12107 | 99.1 | 98.68 | 0.28 | 0.53 | 93.57 | 7.67 | 98.89 | 151 | 78 | 98.9 |
| 141-M | HiSeq 2500 | 11003 | 11898 | 99.10 | 98.68 | 0.28 | 0.51 | 93.52 | 7.70 | 98.89 | 152 | 79 | 98.7 |
| 141-P | HiSeq 2500 | 11416 | 12356 | 99.02 | 98.55 | 0.29 | 0.54 | 93.53 | 7.77 | 98.78 | 154 | 78 | 98.9 |
| 142 | HiSeq 2500 | 11884 | 13261 | 98.32 | 97.71 | 0.37 | 0.88 | 91.43 | 10.77 | 98.01 | 155 | 72 | 98.5 |
| 142-M | HiSeq 2500 | 12302 | 13744 | 98.48 | 98.00 | 0.35 | 0.83 | 91.11 | 11.25 | 98.24 | 160 | 72 | 98.4 |
| 142-P | HiSeq 2500 | 12361 | 13744 | 98.66 | 98.24 | 0.36 | 0.80 | 91.35 | 10.87 | 98.45 | 164 | 73 | 98.5 |
| 143 | HiSeq 2500 | 8778 | 9500 | 99.08 | 98.71 | 0.32 | 0.58 | 93.43 | 8.13 | 98.90 | 125 | 79 | 97.4 |
| 143-M | HiSeq 2500 | 12754 | 13764 | 99.25 | 99.04 | 0.31 | 0.50 | 93.46 | 7.95 | 99.14 | 177 | 78 | 98.4 |
| 143-P | HiSeq 2500 | 11058 | 11911 | 99.24 | 98.88 | 0.31 | 0.60 | 93.72 | 7.77 | 99.06 | 154 | 78 | 98.3 |
| 144 | HiSeq 2500 | 13568 | 15278 | 99.05 | 98.46 | 0.29 | 0.67 | 89.93 | 11.89 | 98.75 | 185 | 73 | 98.6 |
| 144-M | HiSeq 2500 | 10066 | 11369 | 98.95 | 98.43 | 0.30 | 0.68 | 89.71 | 12.10 | 98.69 | 139 | 73 | 97.8 |
| 144-P | HiSeq 2500 | 10484 | 11824 | 98.87 | 98.10 | 0.30 | 0.83 | 90.03 | 11.94 | 98.49 | 144 | 73 | 98.1 |
| 145 | HiSeq 2500 | 11025 | 12051 | 99.15 | 98.79 | 0.29 | 0.63 | 92.44 | 9.16 | 98.97 | 152 | 77 | 98.2 |
| 145-M | HiSeq 2500 | 10599 | 11593 | 99.05 | 98.79 | 0.30 | 0.59 | 92.42 | 9.14 | 98.92 | 148 | 77 | 98.1 |
| 145-P | HiSeq 2500 | 12957 | 14316 | 99.12 | 98.75 | 0.30 | 0.64 | 91.48 | 10.75 | 98.93 | 180 | 76 | 98.6 |
| 146 | HiSeq 2500 | 13039 | 13115 | na | na | na | na | na | 4.67 | 99.42 | 158.97 | 71 | 99.0 |
| 146-M | HiSeq 2500 | 11909 | 11982 | na | na | na | na | na | 4.90 | 99.39 | 146.33 | 70 | 98.0 |
| 146-P | HiSeq 2500 | 13211 | 13313 | na | na | na | na | na | 5.01 | 99.23 | 159.99 | 70 | 99.0 |

[a] Case ID: numbers ending in –M and –P represent the maternal and paternal sample, respectively, of the proband case ID indicated
[b] Unique Aligned (Mbp): the total number of base-pairs in reads that align best to a single location in the reference genome
[c] Total Pass Filter (Mbp): the total number of base-pairs in reads that pass the Illumina quality filters
[d] Avg % Align (PF) Read 1: the average percentage of pass filter base-pairs in Read 1 that align best to a single location in the reference genome
[e] Avg % Align (PF) Read 2: the average percentage of pass filter base-pairs in Read 2 that align best to a single location in the reference genome
[f] Avg % Error rate Read 1: the calculated error rate of bases on Read 1, as determined by aligning to reference genome
[g] Avg % Error rate Read 2: the calculated error rate of bases on Read 2, as determined by aligning to reference genome
[h] Unique-ness %: Percentage of unique reads
[i] Duplicate %: fraction of reads that are identified as duplicate reads – reads whose alignment location is identical to other reads from the same library
[j] Total Reads Aligned: the number of reads that align to the reference genome
[k] Average Coverage: the total number of uniquely aligned bases to the reference genome divided by the size of the reference genome
l Reads hit target/buffer: the number of reads whose alignments overlap either a region targeted by the capture reagent, or the 100bp buffer (or both)
[m] Bases 20+ Coverage: the fraction of bases targeted by the capture reagent that are covered by 20 times or more uniquely aligned reads.
na: not available

**Table S2. Excluded samples without a final report.**

| Sample | Exclusion Category | Exclusion Details | Exome Test Ordered | Sample type | Outcome |
|---|---|---|---|---|---|
| i | Request of referring institution | Family declined (financial burden) | Proband | POC | No testing performed |
| ii | Request of referring institution | Family declined (reason unknown) | Proband | Amniocytes | No testing performed |
| iii | Request of referring institution | Family declined (reason unknown) | Proband | Amniocytes | No testing performed |
| iv | Request of referring institution | Insurance | Standard trio | Amniocytes | Custom gene panel ordered postnatally |
| v | Request of referring institution | Insurance | Proband | POC | No testing performed |
| vi | Request of referring institution | Unknown | Prenatal trio | Amniocytes | No testing performed |
| vii | Insufficient sample | Culture failure (contamination) | Prenatal trio | Amniocytes | No testing performed |
| viii | Insufficient sample | Culture failure (no growth) | Proband | POC | No testing performed |
| ix | Insufficient sample | Insufficient DNA sample received | Proband | DNA (Amnio) | Exome sequencing was performed but failed QC; new sample was requested |
| x | Insufficient sample | Insufficient DNA sample received /Maternal cell contamination | Standard trio | DNA (Amnio) | Exome sequencing was performed but failed QC; new sample was requested |
| xi | Insufficient sample | Insufficient DNA sample received | Proband | DNA (Amnio) | Exome performed postnatally on new sample |
| xii | Insufficient sample | Maternal cell contamination | Proband | DNA (POC) | Fetal testing not pursued. Exome performed on parental samples. |
| xiii | Insufficient sample | Parental samples not received | Standard trio | POC | No testing performed. Authorization not received for revision to proband-only exome. |

Thirteen fetal samples were excluded from the cohort of 146 fetal samples because a final report was not issued. Exclusion details and the final outcome for each case (i.e. whether alternative testing was pursued) demonstrate some of the challenges of exome testing in the prenatal setting.

**Table S3. Incidental findings reported for prenatal exome tests**.

| Case ID | Gene | Variant [RefSeq ID] | Inheritance/Zygosity | Disease Association(s) [MIM#] | Primary molecular diagnosis |
|---|---|---|---|---|---|
| **37-PRE** | *DDX3X* | c.1304T>C (p.L435P) [NM_001193416] | XL/*de novo* het | Mental retardation, X-linked 102 [300958] | none |
| **72-PRE** | *ENG* | c.67+2T>G [NM_000118] | AD/*de novo* mosaic | Telangiectasia, hereditary hemorrhagic, 1 (HHT1) [MIM:187300] | none |
| **87-PRE** | *FBN1* | c.442+1G>A [NM_000138] | AD/het (paternally inherited) | Marfan syndrome [MIM:154700]; MASS syndrome [MIM:604308]; Ectopia lentis, familial [MIM:129600] | *CHRNG* related multiple pterygium syndrome, lethal type [MIM:253290]; Multiple pterygium syndrome, Escobar variant [MIM:265000] |

If a primary molecular diagnosis (i.e. one that is related to the clinical features in the fetus) was reported, that information is listed. Details about primary molecular diagnoses are provided in Table 2.

AD, autosomal dominant; XL, X-linked; het, heterozygous.

**Table S4. Reported fetal phenotypes**.

| HPO term | HPO ID | No. fetuses | (No. molecular diagnoses) |
|---|---|---|---|
| **Abnormality of the head or neck, and/or Abnormality of the ear** | **HP:0000152; HP:0000598** | | |
| Abnormality of the skull | HP:0000929 | 13 | ( 5 ) |
| Abnormal facial shape | HP:0001999 | 13 | ( 2 ) |
| Micrognathia | HP:0000347 | 12 | ( 9 ) |
| Abnormality of the mouth | HP:0000153 | 12 | ( 5 ) |
| Abnormality of the nose | HP:0000366 | 8 | ( 5 ) |
| Abnormality of the outer ear | HP:0000356 | 7 | ( 5 ) |
| Abnormality of globe location | HP:0100886 | 7 | ( 3 ) |
| Retrognathia | HP:0000278 | 5 | ( 1 ) |
| Macrocephaly | HP:0000256 | 5 | ( 0 ) |
| Abnormality of the orbital region | HP:0000494 | 2 | ( 0 ) |
| Abnormality of the neck | HP:0000464 | 1 | ( 0 ) |
| Abnormality of the forehead | HP:0000290 | 1 | ( 0 ) |
| **Abnormality of the skeletal system, and/or Abnormality of the musculature, and/or Abnormality of the limbs** | **HP:0000924 HP:0003011 HP:0040064** | | |
| Abnormality of long bone morphology | HP:0011314 | 23 | ( 12 ) |
| Talipes equinovarus (club foot) | HP:0001762 | 15 | ( 2 ) |
| Flexion contracture | HP:0001371 | 14 | ( 5 ) |
| Aplasia/Hypoplasia of the extremities | HP:0009815 | 8 | ( 4 ) |
| Hand clenching | HP:0001188 | 6 | ( 4 ) |
| Abnormality of the thorax | HP:0000765 | 6 | ( 2 ) |
| Polydactyly | HP:0010442 | 5 | ( 4 ) |
| Abnormality of limbs, unspecified | HP:0040064 | 5 | ( 3 ) |
| Aplasia/Hypoplasia of the ribs | HP:0006712 | 5 | ( 2 ) |
| Aplasia/Hypoplasia involving bones of lower limbs | HP:0006493 | 5 | ( 0 ) |
| Aplasia/Hypoplasia involving bones of upper limbs | HP:0006496 | 5 | ( 0 ) |
| Rocker bottom feet | HP:0001838 | 4 | ( 2 ) |
| Abnormality of vertebral column | HP:0000925 | 4 | ( 1 ) |

| | | | |
|---|---|---|---|
| Skeletal dysplasia | HP:0002652 | 3 | ( 2 ) |
| Abnormality of the foot | HP:0001760 | 3 | ( 1 ) |
| Abnormality of the hand | HP:0001155 | 3 | ( 1 ) |
| Abnormality of vertebrae | HP:0003468 | 3 | ( 0 ) |
| Adducted thumb | HP:0001181 | 2 | ( 2 ) |
| Multiple prenatal fractures | HP:0005855 | 2 | ( 2 ) |
| Overlapping fingers | HP:0010557 | 2 | ( 1 ) |
| Abnormality of the coccyx | HP:0008519 | 2 | ( 1 ) |
| Aplasia/Hypoplasia of toe | HP:0001991 | 2 | ( 0 ) |
| Abnormality of the toes | HP:0001780 | 1 | ( 1 ) |
| Abnormality of the occipital bone | HP:0012294 | 1 | ( 1 ) |
| Abnormality of cartilage | HP:0410007 | 1 | ( 1 ) |
| Aplasia of the fingers | HP:0009380 | 1 | ( 1 ) |
| Split foot | HP:0001839 | 1 | ( 0 ) |
| Abnormality of the fingers | HP:0001167 | 1 | ( 0 ) |
| Aplasia/hypoplasia involving bones of the hand | HP:0005927 | 1 | ( 0 ) |
| Split hand | HP:0001171 | 1 | ( 0 ) |
| Syndactyly | HP:0001159 | 1 | ( 0 ) |
| Abnormal axial skeleton morphology | HP:0009121 | 1 | ( 0 ) |
| Joint dislocation | HP:0001373 | 1 | ( 0 ) |
| Abnormal bone mineral density | HP:0004348 | 1 | ( 0 ) |
| Abnormality of muscle fibers | HP:0004303 | 1 | ( 0 ) |
| **Abnormality of the respiratory system** | **HP:0000765** | | |
| Pleural effusion | HP:0002202 | 10 | ( 5 ) |
| Abnormality of the diaphragm | HP:0000775 | 6 | ( 2 ) |
| Abnormality of the lung | HP:0002088 | 3 | ( 0 ) |
| Aplasia/Hypoplasia of the lungs | HP:0006703 | 2 | ( 2 ) |
| Abnormal lung lobation | HP:0002101 | 1 | ( 0 ) |
| Abnormality of the larynx | HP:0001600 | 1 | ( 0 ) |
| **Miscellaneous abnormalities of prenatal birth and development** | **HP:0001197** | | |
| Intrauterine growth retardation | HP:0001511 | 22 | ( 8 ) |
| Cystic hygroma | HP:0010878 | 20 | ( 10 ) |
| Abnormality of amniotic fluid | HP:0001560 | 19 | ( 6 ) |
| Edema | HP:0000969 | 14 | ( 7 ) |
| Single umbilical artery | HP:0001195 | 12 | ( 3 ) |
| Increased nuchal translucency | HP:0010880 | 11 | ( 4 ) |

| | | | |
|---|---|---|---|
| Hydrops fetalis | HP:0001789 | 10 | ( 4 ) |
| Prenatal movement abnormality | HP:0001557 | 8 | ( 5 ) |
| Abnormalities of placenta or umbilical cord | HP:0001194 | 8 | ( 4 ) |
| Fetal ascites | HP:0001791 | 5 | ( 3 ) |
| Enlarged fetal cisterna magna | HP:0011427 | 3 | ( 2 ) |
| Thickened nuchal skin fold | HP:0000474 | 3 | ( 1 ) |
| Echogenic fetal bowel | HP:0010943 | 2 | ( 1 ) |
| Absence of stomach bubble on fetal sonography | HP:0010963 | 1 | ( 0 ) |
| **Abnormality of the nervous system** | **HP:0000707** | | |
| Abnormality of the cerebral ventricles | HP:0002118 | 23 | ( 5 ) |
| Abnormality of hindbrain morphology | HP:0011282 | 18 | ( 6 ) |
| Aplasia/Hypoplasia of corpus callosum | HP:0007370 | 12 | ( 5 ) |
| Microcephaly | HP:0000252 | 11 | ( 5 ) |
| Hydrocephalus | HP:0000238 | 8 | ( 0 ) |
| Intracranial cystic lesion | HP:0010576 | 6 | ( 3 ) |
| Abnormal cortical gyration | HP:0002536 | 5 | ( 3 ) |
| Abnormality of neuronal migration | HP:0002269 | 5 | ( 0 ) |
| Aplasia/Hypoplasia of cerebrum | HP:0007364 | 4 | ( 2 ) |
| Dandy Walker malformation | HP:0001305 | 4 | ( 1 ) |
| Abnormality of the septum pellucidum | HP:0007375 | 3 | ( 2 ) |
| Abnormality of brainstem morphology | HP:0002363 | 3 | ( 1 ) |
| Abnormality of the spinal cord | HP:0002143 | 3 | ( 0 ) |
| CNS malformation | HP:0000707 | 2 | ( 1 ) |
| Holoprosencephaly | HP:0001360 | 2 | ( 0 ) |
| Encephalocele | HP:0002084 | 2 | ( 0 ) |
| Abnormality of diencephalon | HP:0010662 | 1 | ( 1 ) |
| Fetal dyskinesia | HP:0100660 | 1 | ( 1 ) |
| Cortical dysplasia | HP:0002539 | 1 | ( 1 ) |
| Intracranial hemorrhage | HP:0002170 | 1 | ( 1 ) |
| Hypoplastic olfactory lobes | HP:0006894 | 1 | ( 0 ) |
| Subependymal nodules | HP:0009716 | 1 | ( 0 ) |
| Abnormality of periventricular white matter | HP:0002518 | 1 | ( 0 ) |
| **Abnormality of the cardiovascular system** | **HP:0001626** | | |
| Abnormality of the cardiac septa | HP:0001671 | 18 | ( 7 ) |
| Congenital malformation of the great arteries | HP:0011603 | 9 | ( 0 ) |
| Abnormality of cardiac ventricle | HP:0001713 | 6 | ( 3 ) |

| | | | |
|---|---|---|---|
| Abnormality of the aorta | HP:0001679 | 6 | ( 2 ) |
| Abnormality of the pericardium | HP:0001697 | 5 | ( 2 ) |
| Cardiomyopathy | HP:0001638 | 5 | ( 1 ) |
| Abnormality of the heart valves | HP:0001654 | 3 | ( 2 ) |
| Abnormal anatomic location of the heart | HP:0004307 | 3 | ( 1 ) |
| Echogenic intracardiac focus | HP:0010942 | 2 | ( 1 ) |
| Cardiomegaly | HP:0001640 | 2 | ( 0 ) |
| Abnormality of cardiac atrium | HP:0005120 | 1 | ( 0 ) |
| Abnormality of the pulmonary artery | HP:0004414 | 1 | ( 0 ) |
| Hypoplastic left heart | HP:0004383 | 1 | ( 0 ) |
| Arrhythmia | HP:0011675 | 1 | ( 0 ) |
| **Abnormality of the genitourinary system** | **HP:0000119** | | |
| Abnormal renal morphology | HP:0012210 | 27 | ( 6 ) |
| Abnormality of external genitalia | HP:0000811 | 11 | ( 4 ) |
| Renal cyst | HP:0000107 | 7 | ( 1 ) |
| Abnormality of the urethra | HP:0000795 | 2 | ( 0 ) |
| Abnormal sex determination | HP:0012244 | 1 | ( 1 ) |
| Abnormality of the bladder | HP:0000014 | 1 | ( 0 ) |
| **Abnormality of the abdomen** | **HP:0001438** | | |
| Abnormality of the liver | HP:0001392 | 4 | ( 1 ) |
| Anorectal anomaly | HP:0012732 | 3 | ( 1 ) |
| Abnormality of the stomach | HP:0002577 | 3 | ( 0 ) |
| Abnormality of the abdominal organs | HP:0002012 | 2 | ( 0 ) |
| Omphalocele | HP:0001539 | 2 | ( 0 ) |
| Abnormality of the intestine | HP:0002242 | 1 | ( 0 ) |
| **Other phenotypes** | | | |
| Abnormal eye morphology | HP:0012372 | 4 | ( 1 ) |
| Abnormality of the spleen | HP:0001743 | 4 | ( 0 ) |
| Abnormality of the thymus | HP:0000777 | 1 | ( 0 ) |

The top-branch HPO categories (as shown in Figure 1) are listed. Listed under each top-branch header are the unique HPO-based abnormalities that were reported among the cohort of 146 fetal samples. Many fetuses had more than one anomaly within the same top-branch HPO category, but each applicable top-branch category was only tallied once per fetus.

**Table S5. Pairwise statistical analysis of diagnostic rate based on number of affected organ systems, corrected for multiple comparisons.**

| Pairwise diagnostic rate comparisons (number of top-branch HPO terms per fetus) | P-value (Fisher's exact test) | Bonferroni q-value (critical value = 0.05) | Bonferroni significance |
|---|---|---|---|
| 1 versus >=4 | 0.015 | 0.089 | not significant |
| 1 versus 2 | 0.120 | 0.718 | not significant |
| 1 versus 3 | 0.140 | 0.842 | not significant |
| 2 versus >=4 | 0.492 | 1.000 | not significant |
| 3 versus >=4 | 0.718 | 1.000 | not significant |
| 2 versus 3 | 1 | 1.000 | not significant |

The original p-values based on Fisher's exact test are not corrected for multiple comparisons. The Bonferroni corrected q-values control the family-wise error rate and are not significant. Sample size and diagnostic rates based on the number of affected organ systems are displayed in Figure 1 (main text).

**Table S6. Regions of absence of heterozygosity (AOH) in cases with homozygous variants underlying the molecular diagnosis**.

| Case ID | Gene underlying molecular diagnosis | Homozygous variant underlying molecular diagnosis | Genomic coordinate (GRCh37/hg19) | AOH region containing homozygous variant (approximate) | Sum of AOH regions >5 Mb* (approximate) |
|---|---|---|---|---|---|
| 24-P | GPR126 | c.2677C>T | 6:142636940 | 38 Mb | 269 Mb |
| 46-PRE | PDK1L1 | c.6473+2_6473+3del | 7:47870811 | 4 Mb | NA |
| 60-T | FRMD4A | c.2723C>T | 10:13698866 | 12 Mb | 191 Mb |
| 84-PRE | RAPSN | c.1166+1G>C | 11:47460282 | 7 Mb | 184 Mb |
| 112-PRE | P3H1 | c.12delC | 1:43232630 | 15 Mb | 69 Mb |
| 114-T | IFT80 | c.721G>C | 3:160073857 | 0.5 Mb | NA |

A single nucleotide polymorphism (SNP) array was performed on all fetal samples that underwent exome testing. Regions of AOH greater than 5 Megabases (Mb) were detected across multiple chromosomes for 4 of the 6 samples with molecular diagnoses involving homozygous variants. Case IDs ending in –*PRE* are prenatal trio exomes, those ending in –*T* are standard trio exomes, and those ending in –*P* are proband exomes. NA, not applicable.

**Table S7. Pregnancy outcomes locally referred cases.**

| Case ID | Causal Gene | Disorder | Pregnancy outcome |
|---|---|---|---|
| 90-PRE | COL1A1 | Osteogenesis imperfecta (OI) types 1-4 [MIM:166200, 166210, 259420, 166220]; Caffey disease [MIM:114000]; Ehlers-Danlos syndrome 1 and 7a [MIM:130000,130060] | Alive (at last contact) |
| 6-P | KMT2D | Kabuki syndrome type 1 [MIM:147920] | Stillborn |
| 45-P | KMT2D | Kabuki syndrome type 1 [MIM:147920] | Alive (at last contact) |
| 114-T | IFT80 | Short-rib thoracic dysplasia 2 with or without polydactyly [MIM:611263] | Stillborn |
| 112-PRE | P3H1 | Osteogenesis imperfecta 8 [MIM:610915] | TOP |
| 18-P | RYR1 | Central core disease of muscle [MIM:117000] | TOP |
| 11-P | TMEM67 | Meckel syndrome 3 [MIM:607361]; Joubert syndrome 6 [MIM:610688]; Bardet-Biedl syndrome [MIM:209900]; COACH syndrome [MIM:216360]; Nephronophthisis 11 [MIM:613550] | TOP |

Case ID, Gene, and OMIM disorder, as listed in Table 2 of the main text, are reproduced here for comparison. Case IDs ending in –PRE are prenatal trio exomes, those ending in –T are standard trio exomes, and those ending in –P are proband exomes. TOP= termination of pregnancy.
